# Population Pharmacokinetics of Tideglusib in Congenital and Childhood Myotonic Dystrophy Type 1: Influence of Demographic and Clinical Factors on Systemic Exposure

**DOI:** 10.3390/pharmaceutics17081065

**Published:** 2025-08-16

**Authors:** Alessandro Di Deo, Sean Oosterholt, Joseph Horrigan, Stuart Evans, Alison McMorn, Oscar Della Pasqua

**Affiliations:** 1 Clinical Pharmacology & Therapeutics Group, University College London, London WC1H 9JP, UK; 2 AMO Pharma Ltd., 1 Park Row, Leeds LS1 5AB, UK

**Keywords:** tideglusib, pharmacokinetics, food effect, phase I, myotonic dystrophy, modelling and simulation

## Abstract

**Background**: GSK3β is an intracellular regulatory kinase that is dysregulated in multiple tissues in Type 1 myotonic dystrophy (DM-1). Tideglusib inhibits GSK3β activity in preclinical models of DM-1 and promotes cellular maturation, normalising aberrant molecular and behavioural phenotypes. It is currently in clinical development for the treatment of paediatric and adult patients affected by congenital and juvenile-onset DM-1. Here, we summarise the development of a population pharmacokinetic model and subsequent characterisation of influential demographic and clinical factors on the systemic exposure to tideglusib. The availability of a population PK model will allow further evaluation of age-and weight-related changes in drug disposition, supporting the dose rationale and implementation of a paediatric extrapolation plan. **Methods:** Given the sparse pharmacokinetic sampling scheme in patients receiving tideglusib, model development was implemented in two steps. First, data from Phase I studies in healthy elderly subjects (i.e., 1832 plasma samples, n = 54) were used to describe the population pharmacokinetics of tideglusib in adults. Then, pharmacokinetic model parameter estimates obtained from healthy subjects were used as priors for the evaluation of the disposition of tideglusib in adolescent and adult DM-1 patients (51 plasma samples, n = 16), taking into account demographic and clinical baseline characteristics, as well as food intake. Secondary pharmacokinetic parameters (AUC, C_max_ and T_max_) were derived and summarised by descriptive statistics. **Results:** Tideglusib pharmacokinetics was described by a two-compartment model with first-order elimination and dose-dependent bioavailability. There were no significant differences in disposition parameters between healthy subjects and DM-1 patients. Body weight was a significant covariate on clearance and volume of distribution. Median AUC_(0–12)_ and C_max_ were 1218.1 vs. 3145.7 ng/mL∙h and 513.5 vs. 1170.9 ng/mL, following once daily doses of 400 and 1000 mg tideglusib, respectively. In addition, the time of food intake post-dose or the type of meal appeared to affect the overall exposure to tideglusib. No accumulation, metabolic inhibition, or induction was observed during the treatment period. **Conclusions:** Even though clearance was constant over the dose range between 400 and 1000 mg, a less than proportional increase in systemic exposure appears to be caused by the dose-dependent bioavailability, which reflects the solubility properties of tideglusib. Despite large interindividual variability in the tideglusib concentration vs. time profiles, body weight was the only explanatory covariate for the observed differences. This finding suggests that the use of weight-banded or weight-normalised doses should be considered to ensure comparable exposure across the paediatric population, regardless of age or body weight.

## 1. Introduction

Myotonic dystrophy type 1 (DM-1) is an autosomal dominant, monogenetic disorder caused by expansion repeats of a cytosine-thymine-guanine trinucleotide in the noncoding region of the myotonic dystrophy protein kinase gene on chromosome 19q13.3 [1,2]. Although symptoms most commonly appear in early adulthood, DM-1 can also manifest during childhood (juvenile DM-1) or, in its most severe form, at birth (congenital DM-1), where reduced foetal movements and polyhydramnios can appear already during the prenatal period [3,4,5]. At birth the main features are severe generalised weakness, hypotonia, and respiratory compromise. Surviving infants experience gradual improvement in motor function during the first decade of life, however they may develop typical features of DM-1, including weakness, myotonia, cataracts, and cardiac complications [6,7].

Tideglusib is a brain penetrant orally administered new chemical entity from the thiadiazolidindione chemical family. It acts as a selective adenosyl triphosphate non-competitive inhibitor of glycogen synthase kinase 3 (GSK-3), an enzyme which has fundamental roles for these protein kinases in memory, behaviour, and neuronal fate determination [8,9]. Dysregulation of signalling pathways involving GSK-3 has been associated with several neurological and psychiatric disorders. These findings offered insights into potential therapeutic interventions for DM-1 patients, in whom intellectual disability affects various cognitive domains, including social cognition, memory, language, and visuospatial functioning is also observed [10].

Tideglusib is currently in clinical development for the treatment of adult and paediatric patients affected by congenital or juvenile DM-1. Recently, a Phase II study aimed at the evaluation of the safety and efficacy of tideglusib in adolescent and adult patients (n = 16) has been completed [11]. Whilst this study provided insight into the potential clinical benefits of tideglusib in the target patient population, relatively large variation has been observed in the systemic exposure to tideglusib, which could be associated with its solubility profile, food effect, metabolic polymorphism or individual patient characteristics, such as body weight. However, to date the determinants of intra- and interindividual variability in pharmacokinetics have not been characterised. The aim of the current investigation was therefore to assess the effect of potential covariate factors on the pharmacokinetics of tideglusib using non-linear mixed effects modelling.

Given the limited pharmacokinetic data available from the Phase II study, which relied on sparse blood sampling, a Bayesian meta-analytical approach was used to characterise the pharmacokinetics of tideglusib. First, data from a Phase I study, in healthy elderly subjects, in which a frequent blood sampling scheme was used, were analysed to ensure the identification of the appropriate pharmacokinetic model structure and relevant parameter distributions. Subsequently, parameter estimates and model structure were used as priors for the assessment of the pharmacokinetics in the DM-1 patients along with an allometric function, describing the effect of developmental growth, including age and body weight on the disposition properties of tideglusib. This approach has been successfully used in previous investigations in children [12,13,14,15]. It relies on similar concepts and assumptions applied to historical borrowing, which involves leveraging information from control arms in previous relevant clinical trials, which in turn may further enhance a clinical trial’s efficiency [16,17]. The availability of a population pharmacokinetic model will provide the basis for the dose rationale for younger paediatric patients in future clinical studies. It will also support the implementation of a paediatric extrapolation plan.

## 2. Materials and Methods

### 2.1. Study Population and Study Design

Phase I Study in Elderly Healthy Subjects:

NP031112-07A03 was a double blind, randomised, parallel-group, placebo-controlled, multiple ascending dose study to assess the safety, tolerability, and pharmacokinetics of tideglusib in elderly male and female healthy subjects. The study population consisted of six treatment groups each containing twelve participants, with nine of them randomised to receive tideglusib and three subjects to receive placebo (ratio 3:1). An overview of the inclusion and exclusion criteria of the study is included in the Supplementary Materials.

In total, seventy-two subjects (60 years or older) were allocated into one of the following dose groups and administered 300 mg b.i.d., 400 mg b.i.d., 600 mg q.d., 800 mg q.d., 1000 mg q.d. and 1200 mg q.d tideglusib. Repeat dosing of tideglusib or placebo over 14 days was preceded by single-dose administration followed by a wash-out period of 48 h. The investigational product was administered p.o. as a suspension in water of the formulation F06-037F. Blood samples for the evaluation of tideglusib pharmacokinetics were taken on days 1 and 16 at pre-dose (two samples on day 1), at 20 and 40 min, 1, 1.5, 2, 2.5, 3, 4, 6, 8, 12, 16, 24, 30, 36, and 48 h post-dose. Trough samples were collected pre-dose at days 5, 7, 9, 11, 13, and 15. During dose escalation, progression to the next treatment group was determined following a review of all safety, tolerability, and available exposure data from the previous treatment group. This included assessments of increased levels of ALAT, ASAT, GGT, or alkaline phosphatase exceeding 2.5 times the upper limit of normal (ULN), total bilirubin increases greater than 1.5 times the ULN, or any other safety, tolerability, or exposure concerns. All reported adverse events (AEs) were of mild to moderate severity and transient. No severe AEs or deaths occurred during this study and no SAEs were recorded.

Phase II Study in DM-1 Patients:

AMO-02-MD-2-001 was designed as a Phase II, single-blind, placebo-controlled, fixed-dose study of the safety, pharmacokinetics and efficacy of oral treatment with tideglusib (400 mg or 1000 mg q.d.), in which adolescent and adult patients, aged between 13 and 34 years, with congenital or juvenile onset DM-1 were dosed with tideglusib for 12 weeks [11]. An overview of the inclusion and exclusion criteria can be found in the Supplementary Materials.

Subjects were assigned to either 400 mg or 1000 mg q.d. doses of tideglusib according to a 1:1 ratio. In total, 16 subjects were enrolled in the study. All subjects were treated for 2 weeks with single-blind placebo before starting the single-blind active phase, during which they received the assigned dose and dosing schedule for 12 weeks. Subjects in the 400 and 1000 mg cohorts were combined for the main efficacy analysis. Blood samples for the evaluation of tideglusib pharmacokinetics were taken pre-dose, and between 2- and 4 h post-dose at weeks 2 and 12 after the start of the active treatment. Throughout the study, tideglusib was administered following overnight fasting and food intake was restricted for at least one hour after each daily administration. Compliance with food restriction was monitored through a daily diary. Dosing, blood sampling, and food intake times were recorded for each patient. The primary objective of the study was to investigate the safety and tolerability of tideglusib in this patient population. The secondary objectives were to investigate the pharmacokinetics and efficacy of tideglusib, as assessed by the improvement in clinical endpoints between baseline and end-of-treatment.

### 2.2. Data and Demographic Characteristics

A total of 1832 plasma samples from 54 healthy elderly subjects enrolled in study NP031112-07A03 were included in the pharmacokinetic analysis. The subjects were equally distributed between the six dose groups (nine subjects for each active treatment group). The age of the subjects ranged from 60 to 74 years [mean: 64.3, s.d. = 3.5 years], whereas body weight ranged from 50.7 to 98.1 kg [mean: 74.5, s.d. = 9.9 kg]. Twenty-four subjects were female while 30 were male (Table 1). The observed tideglusib plasma concentration versus time profiles stratified by dose levels are shown in Figure S1 (Supplementary Materials).

From study AMO-02-MD-2-001, a total of 51 evaluable plasma samples from 16 adolescent and adult DM-1 patients were available for the data analysis. Nine samples were found to be below the lower limit of quantification (LLOQ), two of which showed no peak. All these samples were pre-dose (i.e., trough) measurements. As shown in Figure S2 (Supplementary Materials), the observed tideglusib plasma concentration versus time profiles showed large interindividual variability, but with proportionally higher concentrations in patients receiving the 1000 mg q.d. dosing regimen.

The age of patients included in the analysis ranged from 13.8 to 34.9 years [mean: 20.9, s.d. = 5.9 years], whereas body weight ranged from 36.8 to 122.6 kg [mean: 63.6, s.d. = 19.6]. Six of the 16 subjects were female (Table 1).

**Table 1:** Demographic baseline characteristics of the population included in the pharmacokinetic analysis.

	Dose Group (mg)	Dosing Regimen	Subjects (n/Female)	Body Weight (kg)	Height (cm)	Age (Years)
**NP03112-07A03**	300	b.i.d.	9/4	78.0 (62.5–93.7)	171 (158–180)	61 (60–68)
	400	b.i.d.	9/5	77.1 (59.5–98.1)	166 (157–188)	68 (62–72)
	600	q.d.	9/4	74.4 (66.4–88.9)	169.0 (163–182)	64 (60–71)
	800	q.d.	9/5	75.6 (64.2–87.6)	168 (159–182)	65 (61–74)
	1000	q.d.	9/2	72.5 (57.6–87.5)	170 (156–180)	63 (60–65)
	1200	q.d.	9/4	72.7 (50.7–92.7)	167 (155–181)	64 (60–70)
**AMO-02** **MD-2-001**	400	q.d.	8/1	60.3 (36.8–81.4)	168.5 (156–179)	19.4 (13.8–32.6)
	1000	q.d.	8/5	58.7 (45.1–122.6)	160.5 (148–178)	20.8 (17.2–34.9)

Values are summarised by median (minimum–maximum). Summary statistics, including means and standard deviations, are presented in Table S1 (Supplementary Materials).

### 2.3. Bioanalytical Method

The bioanalysis of the human plasma samples was based on a high-performance liquid chromatography with tandem mass spectrometry (HPLC-MS/MS). The method was validated in a previous Phase I study (S01091) and revalidated in Study S29871 in terms of specificity, linearity and intra-assay accuracy and precision, using an UPLC^®^ (Waters) -MS/MS API4000 (AB SCIEX, Marlborough, MA, USA) system. The lower and upper limits of quantification (LLOQ and ULOQ) in human plasma were set at 1 and 2000 ng/mL, respectively. Linearity was evaluated using nine concentrations (spiked plasma samples from calibration standards) ranging between LLOQ and ULOQ. The processing of the chromatograms, the calculation of correlation coefficients (r) and values for the calibration curve slope and intercept were performed using the Analyst^®^ Processing Manager (Analyst^®^ Version 1.4.2, AB SCIEX, Marlborough, MA, USA). The linear regression used a 1/x^2^ weighting factor. The precision of the bioanalytical procedure was evaluated calculating the intra-assay coefficient of variation, expressed as CV%.

### 2.4. Population Pharmacokinetic Modelling

#### 2.4.1. Model Development Using Phase I Adult Data

Given the aims of the current investigation, an initial exploratory analysis included data from three Phase I studies (CL031112-05A01, CL031112-06A02 and NP031112-07A03) in which different formulations (Labrasol^®^/water, F05-052, and F06-037F) were used. These data indicated that formulation differences had an important effect on the pharmacokinetics and overall safety profile of the compound. Labrasol^®^ formulation used in the first two studies was found to be unsuitable for longer studies in animals. In addition, it was also found to produce throat irritation in human volunteers. F05-052 was developed as a powder intended to be orally administered following reconstitution in water. In study NP031112-07A03 formulation F06-037F, an evolution of the F05-052 formulation, demonstrated better dispersibility and organoleptic properties. This finding led to further assessment of its pharmaceutical properties and subsequent development for future clinical trials with tideglusib. Consequently, only data from study NP031112-07A03 were used for the development of a population pharmacokinetic model for tideglusib.

Pharmacokinetic modelling was implemented using a non-linear mixed effects approach. First, a base model was built testing different compartments to describe the observed disposition characteristics. Then, the appropriate stochastic components describing the inter-individual variability were identified and added to the base model. Inter-individual variability in pharmacokinetic parameters was assumed to be log-normally distributed. The residual error was characterised using a proportional error model.

Given the anticipated use of the model as a prior for parameter estimation in subsequent studies including sparse blood sampling in paediatric and/or adult subjects, model development was based on allometric principles, assuming body weight as a significant covariate factor on the pharmacokinetics of tideglusib. This effect was parameterised using a reference body weight of 70 kg and fixed allometric exponents on clearance (0.75) and volumes (1). Even so, a covariate analysis was implemented to explore measurable sources of variability in the pharmacokinetics of tideglusib. In addition to body weight, several factors were considered (e.g., age, BMI, dose, and food intake, i.e., fasting or fed state). In situations in which collinearity exists between covariates, such as age and weight, the choice of covariates to be selected was guided by a careful review of the factors that are most likely to be the primary cause of interindividual differences in pharmacokinetic parameters. A standard forward inclusion backward deletion procedure was used to select the covariates [18]. Statistically significant covariates as well as those considered clinically relevant were added to the base model, with one covariate into one parameter at a time.

Briefly, a comparison between hierarchical models was based on the difference in −2 log likelihood, which is assumed to be approximately χ^2^-distributed, with degrees of freedom equal to the difference in the number of parameters between two tested models. A reduction in the OFV ≥ 3.84, χ^2^ < 0.05 for 1 degree of freedom (df) using the FOCE-I estimation method was considered statistically significant. The significance test as well as the inclusion steps were repeated until all significant covariates were evaluated, and the final model was defined. During the final modelling steps, only the covariates which resulted in an OFV reduction of ≥7.88 (*p* < 0.005) were kept in the model. After the full model was identified, the significance of each covariate was tested individually by removal one at a time from the full model (backward deletion). A covariate was retained in the model if, upon removal, the OFV increased by more than 6.64 points (χ^2^ < 0.01 1 df) [18].

#### 2.4.2. Parameter Estimation in DM-1 Patients

**Figure 1:**
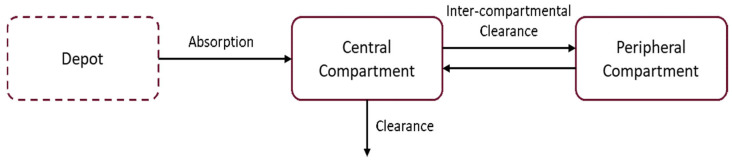
Schematic representation of the pharmacokinetic model structure of tideglusib in elderly healthy subjects. Tideglusib pharmacokinetics was described by a two-compartment model with first-order elimination and dose-dependent bioavailability. The full model included additional terms describing the effect of body weight as a covariate on clearance (CL), intercompartmental clearance (Q), and volumes of distribution (V2 and V3), as well as a dose-dependent effect on the absorption rate constant (KA) and bioavailability (F1).

The use of a rich sampling scheme in study NP031112-07A03 has allowed the identification of the model structure along with relevant parameter distributions describing the inter-and intraindividual variability in pharmacokinetic parameters (Figure 1). These estimates were subsequently used as prior information for the characterisation of the systemic exposure in DM-1 patients. The analysis was performed according to the $PRIOR NWPRI subroutines in NONMEM. When using NWPRI, fixed-effect parameters are assumed to be normally distributed, whereas the random effects (ω^2^ and σ^2^) are assumed to be described by an inverse Wishart distribution [19,20].

Both informative and non-informative priors have been tested. Informative prior distributions for fixed-effect parameters were implemented using the full variance-covariance matrix from the pharmacokinetic model in healthy elderly subjects. For informative priors on random effects, the degree of freedom was calculated with the formula:

df = 2∙[ω^2^/(SE of ω^2^)]^2^ + 1 (1)

Empirical Bayesian post hoc estimates were then derived for DM-1 patients.

#### 2.4.3. Model Evaluation

Model performance was assessed by numerical, graphical, and statistical procedures, including standard goodness-of-fit plots, visual predictive check (VPC), bootstrapping, normalised prediction discrepancy error (NPDE) and mirror plots. In detail, goodness-of-fit was assessed by graphical methods, including population and individual predicted vs. observed concentrations, conditional weighted residuals vs. observed concentration or time, correlation between parameters. Further evaluation of the variance-covariance structure and overall random effects in the model was based on NPDE diagnostics [21].

VPCs were used to evaluate the adequacy of the final model parameter estimates, including the effects of statistically significant covariates [22]. One thousand replicates of the original data set were simulated, and the 90% prediction interval was computed. The observed concentration versus time data were plotted along with the prediction intervals to visually assess the concordance between predicted and observed data.

Bootstrapping was performed to assess bias, model stability, generate standard errors and confidence intervals for the parameter estimates [23]. For bootstrapping, 1000 new data sets were generated by sampling individuals with replacement from the original data set and then fitting the model to each new data set [24].

Subsequently, to generate mirror plots, the parameter estimates of the final model were used to simulate plasma concentrations in patients with similar demographic characteristics, dosing regimens, and sampling schemes as those in the original clinical study. Mirror plots of individual predicted versus observed concentration were created to evaluate the variance-covariance structure, which can be assessed by the degree of similarity between the original fit and the pattern obtained from the simulated datasets.

The same model validation procedures have been applied for the analysis of the pharmacokinetic data in healthy adult elderly subjects and DM-1 patients.

### 2.5. Secondary Pharmacokinetic Parameters

The final parameter estimates were used to simulate individual concentration vs. time profiles based on 6 min sampling intervals. Secondary metrics of exposure were then derived by non-compartmental methods, namely: peak concentration (C_max_), trough concentration (C_min_), area under the concentration vs. time curve (AUC_(0–24)_ or AUC_(0–12)_), time at which peak concentration occurs (T_max_), elimination half-life (T_1/2_) and average steady-state concentration (C_ss_).

### 2.6. Exploratory Analysis of the Effect of Food Intake on the Systemic Exposure in DM-1 Patients

Throughout the Phase II clinical study (AMO-02-MD-2-001), tideglusib was administered to DM-1 patients following overnight fasting. Food intake was then restricted for at least one hour after each administration. Food intake times as well as type of meal were recorded, according to the following categories: light, standard or other.

In total, 30 evaluable records, collected from two different visits were used for the purpose of this analysis. Thus, to explore the effect of food on the extent and rate of absorption of tideglusib, AUC_(0–12)_, AUC_(0–24)_, and C_max_ values on visit 4 and visit 9 were compared by stratifying the results by meal type. Results were summarised by median and range.

### 2.7. Effect of Body Weight on the Pharmacokinetics of Tideglusib and Dose Rationale for the Paediatric Population

Simulation scenarios were implemented to explore the implications of age/weight-related changes in the systemic exposure to tideglusib. As there is limited information *in vivo* on the metabolism of tideglusib, scaling of disposition parameters was based on allometric principles, with total body weight as the primary determinant of changes in pharmacokinetics.

Given the importance of minimising dispensing errors and ensuring a child-friendly dosing regimen, fixed dose weight-banded regimens were prioritised during this evaluation. Scenarios consisted of a virtual population of 71 patients per weight band, and dose level, considering a uniform distribution of weight ranging from 5 to 75 kg. For each dose group, 1000 iterations have been performed. Simulated steady state profiles were generated using a rich sampling scheme (every 0.1 h) with fixed dosing intervals (once daily, every 24 h).

The adequacy of the proposed dosages has been evaluated by comparing the predicted exposure measures, AUC_0–24_ and C_max_, with the corresponding reference values in adult subjects with a body weight between 70 and 75 kg receiving 1000 mg dose of tideglusib under fasting conditions. Reference values [median (90% prediction interval)] were 2560.8 (1282.5–5286.7) ng/mL∙h and 929.6 (446.4–1834.4) ng/mL for AUC_0–24_ and C_max_, respectively. In addition to summary statistics, the proportion of subjects exceeding the 95th percentile of the exposure range in adults was also calculated. Even though a maturation function cannot be defined in absence of further data, the proposed dosing regimen does take into account the implications of interindividual variability and time-dependent changes in metabolic clearance (i.e., CYP3A4 metabolism, as initially suggested by *in vitro* data). It also considers dose-dependent solubility and its effect on the bioavailability of tideglusib.

### 2.8. Software

Clinical and demographic baseline data as well as dose and drug concentrations were imported into R (version 4.4.0, R Core Team, 2021) [25] for exploratory analysis and formatted for subsequent modelling and simulation in NONMEM version 7.5.1 (ICON Development Solutions, Baltimore, MD, USA). The analysis was conducted in combination with PsN 5.3.1. All required data manipulation, including graphical and statistical summaries were performed in R.

## 3. Results

### 3.1. Tideglusib Pharmacokinetics in Elderly Healthy Subjects and DM-1 Patients

**Figure 2:**
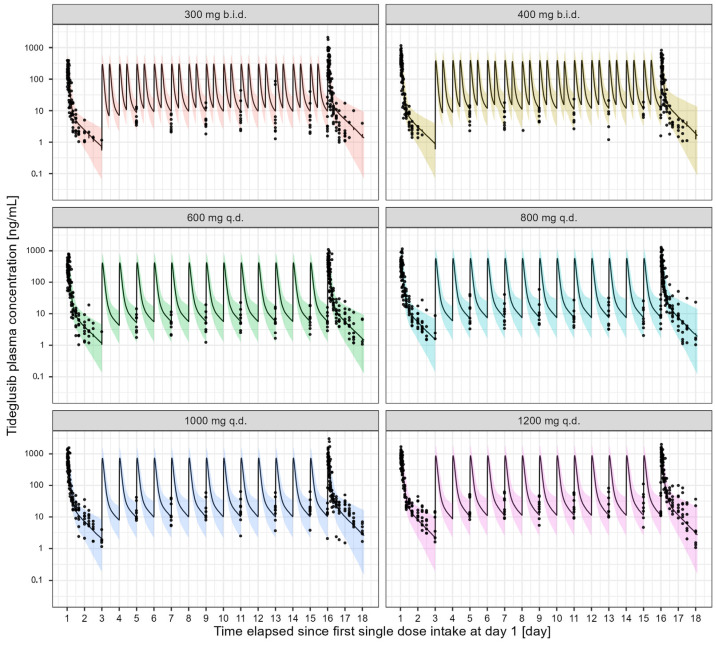
Visual predictive checks describing the performance of the final model of tideglusib in elderly healthy subjects (n = 54), stratified by dose level. The solid circles are the observed concentrations; solid black lines represent the median of the simulated data. The shaded areas depict the 90% prediction intervals for the simulated profiles.

Tideglusib pharmacokinetics in elderly healthy subjects was adequately described by a 2-compartment model. Despite linear pharmacokinetics, the concentration vs. time profiles of tideglusib were found to be dose-dependent, with systemic exposure probably being affected by the limited solubility of the compound. This phenomenon was described by the different estimates for the first-order absorption rate constant (KA) and relative bioavailability (F1). Weight was the only covariate to have a significant effect on CL, V2, V3 and Q. In addition, interindividual variability (IIV) was estimated for CL, V2, V3, Q and KA. Residual variability was parameterised using a proportional error model (Table 2), but a separate parameter was required for doses lower and higher than 400 mg. All parameters were well estimated without significant correlations between them. Fixed-effect parameters were estimated with good precision (%RSE < 27.4%) as were the inter-individual variabilities (%RSE < 41.1%). The VPC (Figure 2) showed that the observed tideglusib concentrations fall within the 90% confidence interval of the simulated values for oral dosing. The non-parametric bootstrap estimates of the model parameters indicated model stability, with acceptable confidence intervals for all parameter estimates.

The diagnostic plots did not reveal any bias or significant deviation between predicted and observed data. This was corroborated by the NPDE plots, which displayed normally distributed prediction errors and did not show any specific bias in model predictions (Supplementary Materials, Figures S3–S5). The final model was therefore deemed to have acceptable predictive performance to be used as priors in conjunction with sparse blood sampling (Table 2).

**Figure 3:**
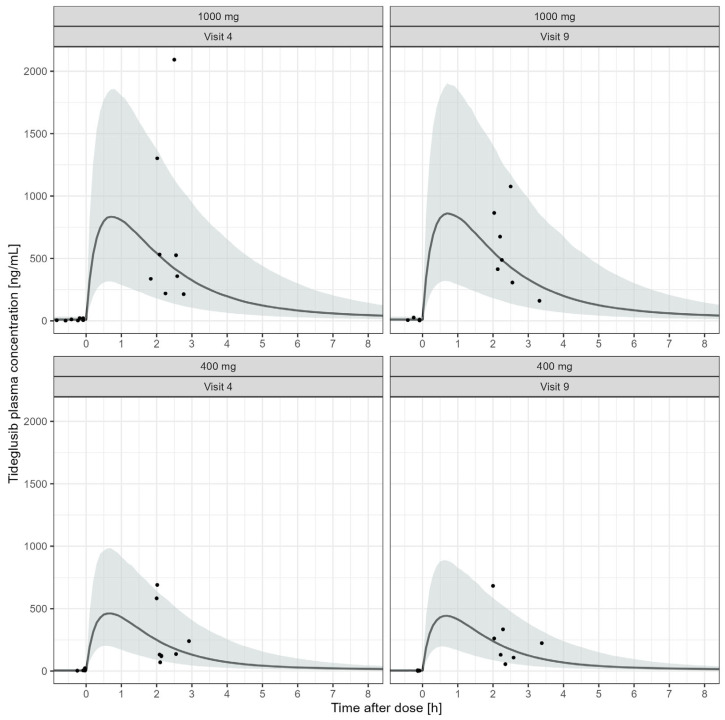
Visual predictive check for the final model of tideglusib in DM-1 patients (n = 16). The black circles are observed concentrations, and the solid black line represents the median of the simulated data. The shaded area indicates the 90% prediction interval of the simulated profiles. Data are stratified by dose group and treatment visit. Time in the *x*-axis is the time after the last dose. Observed concentrations at or before t = 0 are trough samples collected prior to the last dose.

In fact, tideglusib exposure in adolescent and adult patients with congenital and juvenile-onset DM-1 was adequately described by the model. Similarly to the differences observed in healthy subjects, dose (strength) was found to have a statistically significant effect on residual variability in the patient population, with different parameter estimates for doses higher than 400 mg. Weight was the only covariate to have a significant effect on the disposition of tideglusib. All parameters were well estimated with good precision (%RSE < 23%) as were the IIV (%RSE < 32%). A VPC (Figure 3) shows that the observed tideglusib concentrations fall within the 90% prediction interval. The predictive accuracy and the stability of the final model is corroborated by NDPE and mirror plots. Finally, diagnostic plots indicate that the model adequately describes the data and produces unbiased population and individual predictions (Supplementary Materials, Figures S6–S11). Therefore, the final model was deemed to have acceptable predictive performance for simulation purposes.

### 3.2. Secondary PK Parameters

Using the final population model, predicted plasma concentration vs. time profiles were derived using a serial sampling scheme. Median estimates for adolescent and adult DM-1 patients receiving doses of 400 mg and 1000 mg were, respectively, 1218.1 and 3145.7 ng/mL∙h for AUC_0–12_ and 513.5 and 1170.9 ng/mL for C_max_. The median elimination half-life (T_1/2_) ranged between 1.7 h and 2.0 h.

These results show that administration of fixed doses irrespective of interindividual differences in body weight leads to higher exposure in the younger subjects, who have lower body weight. The summary statistics for the secondary pharmacokinetic parameters are shown in Table 3.

### 3.3. Influence of Type and Time of Food Intake on the Systemic Exposure to Tideglusib

**Figure 4:**
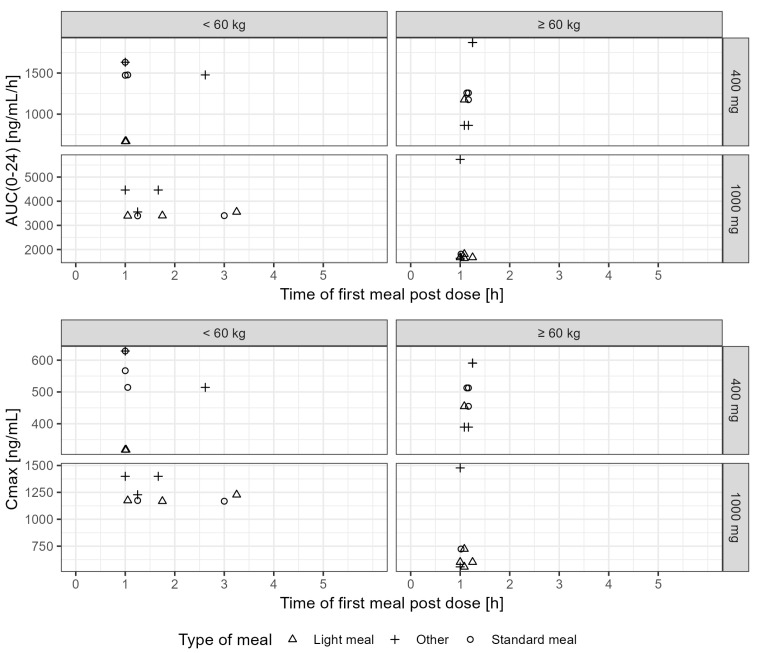
Effect of type of meal on exposure to tideglusib administration in DM-1 patients. Upper and bottom panels show, respectively, AUC_(0–24)_ and C_max_ vs. the time of the first meal post dose. Data are stratified per dose level (rows) and into two body weight groups, i.e., below or above the population median (60 kg). Points depict the individual predicted parameter values. Each meal type is depicted by a different symbol.

In the 400 mg dosing group, three patients had a standard meal, seven had a light meal and five had another type of meal. In the 1000 mg dosing group, six patients had a standard meal while three had a light meal and six had other types of meals. Food intake took place at different times after drug administration under fasting conditions. There was no evidence of an effect of food or meal type on the predicted exposure to tideglusib. As shown in Figure 4, there are no clear differences in AUC and C_max_ across dose levels for the different meal types. The intake of food at different times after dosing does not seem to affect systemic exposure; no trends are observed even after stratification of the data by body weight. In Table 4, the secondary PK parameters of interest, AUC_(0–24)_, AUC_(0–12)_ and C_max_ are stratified by meal type.

### 3.4. Influence of Body Weight on Systemic Exposure to Tideglusib

**Figure 5:**
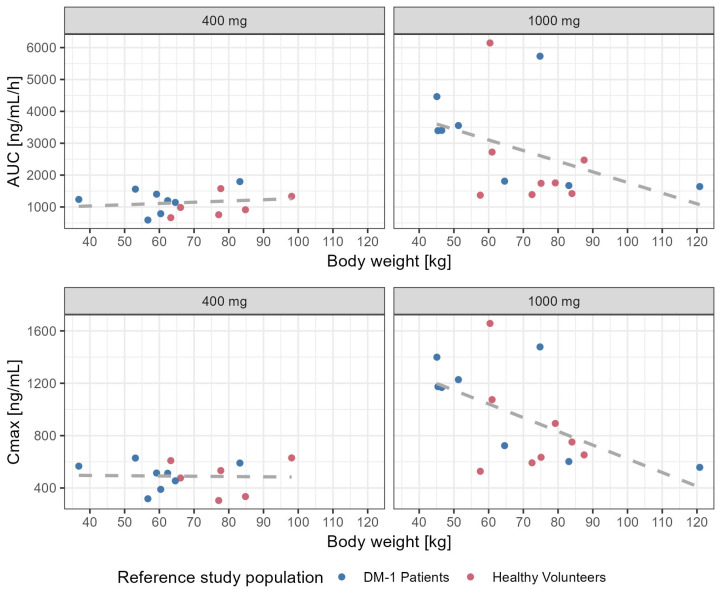
Effect of body weight on systemic exposure (AUC) and peak concentration C_max_) of tideglusib in elderly healthy subjects and DM-1 patients. Solid circles depict the predicted values while dashed lines are a general linear function describing the trends in the data.

Body weight was identified as a covariate on the clearance and volume of distribution of tideglusib. This effect is also observed when exploring the correlation between secondary pharmacokinetic parameters and body weight in both healthy elderly subjects and adolescent and adult DM-1 patients. Despite the relatively large interindividual variability, it seems that higher exposure is expected to occur in subjects with lower body weight (Figure 5).

### 3.5. Dose Rationale for Paediatric Patients

The final pharmacokinetic model in DM-1 patients was used to support prediction and extrapolation of the pharmacokinetic properties of tideglusib across different age groups, assuming that the exposures observed in adults and adolescents following 1000 mg q.d. dose are clinically relevant and can be considered as a target for efficacy. Based on the putative mechanism of action of tideglusib, the approach also assumes a similar pharmacokinetic-pharmacodynamic relationship for tideglusib in adults and children affected by DM-1, despite the heterogeneity in the onset and clinical presentation of the condition in children. Simulation scenarios enabled the identification of suitable dosing regimens that maximise the proportion of subjects who achieve the observed exposure range in adult and adolescent patients receiving 1000 mg dose of tideglusib (Table 5). This scenario provided the highest proportion of patients meeting the target exposure range.

**Figure 6:**
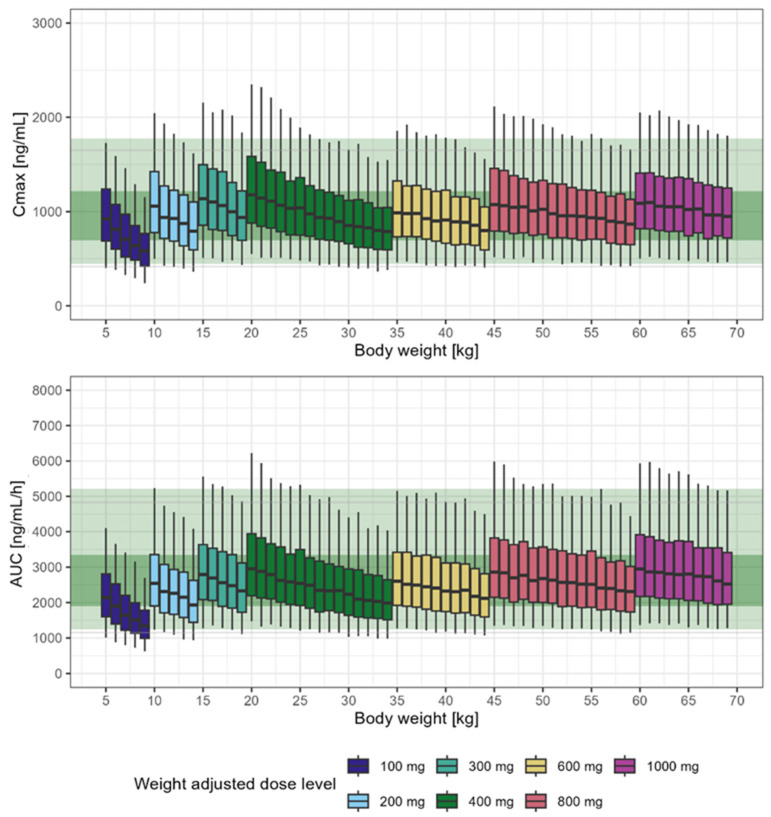
Predicted C_max_ (upper panel) and AUC_0–24_ (lower panel) in children and adults stratified by bodyweight. Shaded green area depicts the reference exposure range (5th–95th and 25th–75th percentiles) predicted in adults with body weight equal to 70–75 kg and receiving 1000 mg. The horizontal line in the whisker-box plots represents the median, bottom and top edge of the box are the 25th and 75th percentiles. The vertical line (whiskers) represents the 5th and 95th percentiles of the data distribution. Each box refers to a specific weight group.

Figure 6 depicts, the predicted AUC_0–24_ and C_max_ in a paediatric population stratified by dose and body weight. These results are summarised per median (90% prediction intervals) and mean (±SD) in the Supplementary Tables S2 and S3. For completeness, to address potential tolerability concerns in the youngest subjects, a weight-banded regimen has been explored considering the 400 mg as the reference dose in adults (Table S4). This scenario could be implemented as a run-in phase at the start of treatment prior to the use of a higher (maintenance) dose. The results from this simulation scenario are summarised in the Supplementary Materials Tables S5 and S6 and presented graphically in Figure S12.

Whilst the current modelling and simulation study relies primarily on allometric principles, the age/weight-related changes in exposure reflect the well-established role of developmental growth (size and organ function) on drug disposition. Implementation of a protocol including interim steps and protocol inclusion based on a staggered approach would allow assessment of potential age-related changes, and further refinement of the recommended weight bands and doses for children affected by congenital and juvenile onset DM1.

## 4. Discussion

Under the EU Paediatric Regulation and US Best Pharmaceuticals for Children Act (BPCA) and the Pediatric Research Equity Act (PREA) amendments of 2007, pharmaceutical companies are expected to conduct clinical studies in the paediatric population. Evidence from these studies does not only support the regulatory approval of the medicinal product but also provides the basis for prescribing information.

Whilst evidence generation in paediatric diseases with relatively high incidence and prevalence should not represent a challenge for the implementation of a paediatric development plan, this is not the case for orphan drugs developed for rare diseases. As a matter of fact, four main issues thwart the drug development process: 1. poor diagnostics and assessment of disease progression; 2. the large heterogeneity in disease pathophysiology and treatment effects; 3. lack of treatment-sensitive endpoints and biomarkers to predict and quantify treatment outcomes, and 4. paucity of patients and data availability to draw robust conclusions regarding the efficacy and safety profile of a medicinal product based on standard statistical approaches. Our investigation focuses on the latter point, highlighting the application of modelling, simulation and extrapolation approaches as a tool to maximise the use of data, thereby optimising evidence generation during the early stage of development of an orphan drug [26,27,28]. In a setting in which the lack of data constitutes the main burden to be overcome, the use of quantitative clinical pharmacology concepts allow the implementation of the learning vs. confirming paradigm, providing insight into issues that are critical for the implementation of conclusive study protocols, such as the dose rationale, and understanding of the determinants of variability in drug exposure [29,30].

As shown in the current study, the availability of Phase I data in elderly healthy subjects provided the basis for the characterisation of the population pharmacokinetics of tideglusib. These data allowed for parameter estimates describing the absorption, distribution and elimination processes with adequate accuracy and precision. Parameter estimates were subsequently used as priors for the assessment of systemic exposure in DM-1 patients, taking into account the effect of body weight as an important covariate affecting the disposition characteristics of tideglusib. This effect has been identified for most drugs used in children and reflects the effect of developmental growth on metabolic processes [13,26]. Eventually, this effect can be parameterised together with a maturation function, describing the impact of ontogeny on drug biotransformation (clearance) [12,13,14,15].

The main finding from our model is that tideglusib shows dose-dependent bioavailability across the dose range evaluated in healthy elderly subjects as well as in congenital and juvenile onset DM-1 patients. This effect is likely to be caused by the limited solubility of the compound, even though other factors, such as variable first-pass metabolism may also play a role. Our parameterisation suggests that oral bioavailability decreases with increasing doses, even though we have not been able to quantify these changes in a continuous manner. Instead, bioavailability and absorption rate constant were dichotomized into two levels, with exposure data following a 400 mg dose as reference. Accurate description of the individual concentration vs. time profiles at higher dose levels required the use of lower estimates for the oral bioavailability and absorption rate constant.

From a pharmacokinetic perspective, there was no accumulation, metabolic inhibition, or induction observed during treatment. Despite the evidence of dose-dependent bioavailability, the population estimate for the apparent volume of distribution at steady state (i.e., sum of the volumes of distribution for the central and peripheral compartments) was 1140 L, which suggests that tideglusib distributes into tissues well beyond total body water. Mean population estimates for the apparent systemic clearance of 341 L/h likely indicate drug elimination taking place at a rate close to the hepatic blood flow [31]. In addition, whilst no formal comparison or pooled analysis was performed including healthy elderly subjects and congenital and juvenile onset DM-1 patients, model parameter estimates reveal that the disease does not affect the pharmacokinetics of tideglusib. This is in line with previous reports indicating no significant disease-related changes in hepatic metabolism, haemodynamics or vascular permeability [31].

Time of food intake post dose did not affect the pharmacokinetics of tideglusib, suggesting a favourable safety profile of tideglusib in terms of altered liver enzymes when administered using the selected F06-037F formulation. Thus, in addition to its better dispersibility and organoleptic properties compared to the previous dosage forms, these findings confirm the decision to use this formulation in the subsequent development in DM-1 patients. Indeed, in studies CL031112-05A01 and CL031112-06A02 a marked effect of food was observed in healthy young and elderly volunteers dosed with 200 mg or 400 mg of formulation F05-052 following a high-fat or a normal breakfast within 30 min before drug administration. In detail, plasma levels were substantially increased, and peak concentrations were delayed in fed conditions. Administration with a meal also resulted in a higher frequency of transaminase elevations than administration in fasting conditions. Abnormalities in serum transaminases have been observed, particularly in a twice-daily dosing regimens, when the drug was administered with food and at the highest doses. However, it is worth noting that the increase in liver enzymes observed with previous formulations may be associated with GSK-3 β inhibition for the induction of liver repairing and regeneration process. Evidence from in vitro protocols shows that numerous GSK-3 β inhibitors (LY 2090314, AR-A014418, tideglusib, solasodine, CHIR99021, 9-ING-41, SB-216763) play an important role in stimulating the liver regeneration process through Wnt/β-catenin signalling pathway [31]. Given the chronic nature of this treatment, further evaluation of the administration of lower doses of tideglusib with food should be considered. An exposure range comparable to that observed following administration of tideglusib under fasting conditions may be achieved with the use of lower doses with food. Slower gastric emptying may also allow for lower peak concentrations.

The identification of body weight as a covariate on the parameters describing the disposition of tideglusib is not unexpected, as such an effect is often observed with drugs showing similar distributional properties, especially when considering adolescents or other groups with different demographic characteristics [32,33,34]. In fact, the use of allometric principles to extrapolate pharmacokinetics from adult subjects to children is widely accepted [32,33,34]. Based on the available data, no other source of variability has been identified that would further contribute to improving model performance and consequently support the optimisation of the dose level to be used in future trials.

We acknowledge some limitations of our investigation. First, it should be recognised that as there is incomplete data on the primary route of metabolism in vivo, neither a maturation function nor an age-dependent allometric exponent were included in the simulated scenarios [32,33,34]. In addition, given potential safety concerns, the magnitude of food-drug interaction has not been characterised in the target population. Our approach focused therefore on the time window of food intake after drug administration. Similarly, we have not been able to retrieve detailed information on meal caloric content, which prevented us from exploring these factors as potential covariates. Consequently, they were excluded from the dosing optimisation process, with the assumption that all individuals were in a fasting state. Nevertheless, the use of priors based on the pharmacokinetics in adults has been successfully implemented for several products [32,33,34]. It has proved to be very effective in a situation that reflects early clinical development of drugs for rare diseases, such as transfusion-dependent haemoglobinopathies, anthrax infection, eosinophilic oesophagitis [32,33,34].

In conclusion, tideglusib shows dose-dependent bioavailability, with systemic exposure increasing less than proportionally with increasing doses. Despite the relatively high intra- and interindividual variability in plasma concentrations, body weight was found to be a covariate on clearance and volume of distribution, with higher AUCs and C_max_ values observed in subjects with decreasing body weight. Moreover, the availability of a population pharmacokinetic model allowed for extrapolation and prediction of the pharmacokinetics in the paediatric population, supporting the dose rationale for future clinical trials in children affected by myotonic dystrophy.

## Figures and Tables

**Table 2:** Final population pharmacokinetic parameter estimates for tideglusib in healthy elderly subjects and DM-1 patients.

	AMO-02-MD-2-001			NP031112-07A03		
Parameter (Unit)	Population Estimate	RSE (%)	Bootstrap Median (5th–95th Percentiles)	Population Estimate	RSE (%)	Bootstrap Median ^a^ (5th–95th Percentiles)
Clearance, CL (L/h)	341	12.4	336 (268–389)	327	17.0	333 (260–408)
Volume of distribution central compartment, V2 (L)	154	16.4	151 (108–183)	152	27.4	156 (93–239)
Absorption rate constant, kA (1/h)	0.767	6.8	0.760 (0.677–0.828)	0.78	11.3	0.78 (0.67–1.09)
Absorption rate constant, kA (dose > 400 mg) (1/h)	0.609	1.6	0.608 (0.589–0.623)	0.61	6.6	0.61 (0.55–0.69)
Intercompartmental clearance, Q (L/h)	66.1	22.1	64.6 (41.9–82.2)	66.1	26.0	67.0 (46.8–94.1)
Volume of distribution peripheral compartment, V3 (L)	986	18.3	971 (692–1180)	1010	21.1	1021 (759–1375)
Bioavailability (doses ≤ 400 mg)	1	FIXED	FIXED	1	FIXED	FIXED
Bioavailability (doses > 400 mg)	0.88	13.2	0.87 (0.68–1.01)	0.85	18.8	0.86 (0.64–1.11)
**Interindividual Variability**	**Population Estimate (CV%) ^b^**	**RSE** **(%)**	**Bootstrap Median** **(5th–95th Percentiles)**	**Population** **Estimate (CV%) ^b^**	**RSE** **(%)**	**Bootstrap Median ^a^** **(5th–95th Percentiles)**
ηCL variance	0.186 (43.1)	22.7	0.18 (0.13–0.27)	0.18 (43.5)	16.9	0.18 (0.12–0.24)
ηQ variance	1.15 (107.2)	31.6	1.09 (0.71–1.83)	0.96 (98.2)	23.5	0.93 (0.61–1.31)
ηV3 variance	1.01 (100.5)	28.6	0.96 (0.67–1.57)	0.90 (95.1)	22.2	0.89 (0.59–1.24)
ηV2 variance	0.321 (56.7)	3.1	0.32 (0.31–0.34)	0.33 (57.8)	36.5	0.32 (0.15–0.58)
ηKA variance	0.069 (26.4)	2.2	0.07 (0.07–0.07)	0.06 (26.2)	41.1	0.06 (0.02–0.11)
**Residual Error**	**Population Estimate**	**RSE** **(%)**	**Bootstrap Median** **(5th–95th Percentiles)**	**Population Estimate**	**RSE** **(%)**	**Bootstrap Median ^a^** **(5th–95th Percentiles)**
Proportional error (doses ≤ 400 mg)	0.54	1.8	0.54 (0.53–0.56)	0.56	7.2	0.55 (0.49–0.62)
Proportional error (doses > 400 mg)	0.46	0.9	0.46 (0.45–0.47)	0.45	4.0	0.45 (0.42–0.48)

Abbreviations: CV = coefficient of variation, RSE = percent relative standard error, θ = PK fixed-effect parameter; η = inter-individual variability; Ω = variance of inter-individual variability in population PK parameter; σ = residual variance. ^a^: Non-parametric bootstrap results are presented as median and 5th–95th percentiles. Bootstrapping was based on re-sampling of 1500 data sets from the original data set and subsequent re-estimation using the final model. ^b^: Coefficient of variations have been calculated as: $CV=sqrt\left(exp\left({Ω}^{2}\right)\right)\times 100$.

**Table 3:** Statistical summary of the secondary pharmacokinetic parameters of tideglusib in elderly healthy subjects (n = 54) and in adolescent and adult DM-1 patients (n = 16).

Study	Dose (mg)	C_max_ (ng/mL)	C_min_ (ng/mL)	C_SS_ (ng/mL)	T_max_ (h)	T_1/2 _ (h)	AUC_(0–12)_ (ng/mL∙h)
**NP03112-07A03 ^a^**	400 b.i.d.	504.7 (312.0–624.9)	9.8 (7.2–22.4)	79.2 (57.3–126.4)	0.7 (0.4–1.0)	1.5 (1.0–2.8)	949.7 (687.9–1516.1)
	1000 q.d.	702.0 (550.3–1453.3)	18.5 (7.0–37.8)	78.3 (59.4–219.0	0.7 (0.3–1.3)	4.0 (1.8–6.7)	1749.8 (1376.7–4947.7)
**AMO-02** **MD-2-001 ^b^**	400 q.d.	513.5 (342.9–615.4)	4.1 (2.1–11.3)	56.9 (30.7–74.4)	0.75 (0.3–1.0)	2.0 (1.3–5.4)	1218.1 (660.3–1713.8)
	1000 q.d.	1170.9 (573.3–1450.1)	5.6 (3.4–16.2)	141.6 (68.8–220.3)	0.7 (0.6–1.2)	1.7 (1.0–2.5)	3145.7 (1571.4–5109.4)

Data are medians and (5th–95th percentiles). ^a^ For study NP03112-07A03, secondary pharmacokinetic parameters have been derived at day 16. ^b^ For study AMO-02-MD-2-001, secondary pharmacokinetic parameters have been derived from Visit 4.

**Table 4:** Statistical summary of the secondary pharmacokinetic parameters per type of meal.

	Dose (mg)	Weight (kg)	n	C_max_ (ng/mL)	AUC_(0–12)_ (ng/mL·h)	AUC_(0–24)_ (ng/mL·h)
**Light Meal**	400	<60	1	317.9	590.8	669.2
		≥60	1	454.8	1143.5	1175.8
	1000	<60	3	1174.1 (1167.8–1227.8)	3153.4 (3138.1–3462.7)	3402.9 (3394.4–3555.8)
		≥60	3	602.1 (557.8–723.6)	1586.6 (1563.2–1743.7)	1672.7 (1640.7–1810)
**Standard Meal**	400	<60	3	566.8 (514.3–628.7)	1402.3 (1237.3–1560.0)	1477.2 (1473.0–1631.5)
		≥60	2	483.8 (454.8–512.7)	1171.3 (1143.5–1199.1)	1216.9 (1175.7–1258.1)
	1000	<60	2	1170.9 (1167.8–1174.1)	3145.8 (3138.1–3153.4)	3398.7 (3394.5–3402.9)
		≥60	1	723.6	1743.8	1810.0
**Other Meal**	400	<60	2	571.5 (514.3–628.7)	1481.1 (1402.3–1560.0)	1554.3 (1477.2–1631.5)
		≥60	2	490.1 (389.4–590.8)	1293.1 (789.4–1796.7)	1367.3 (863.5–1871.2)
	1000	<60	2	1313.5 (1227.8–1399.2)	3817.5 (3462.7–4172.4)	4009.4 (3555.8–4463.0)
		≥60	2	1017.7 (557.8–1477.6)	3588.6 (1563.2–5614)	3685.8 (1640.7–5730.8)

Data are summarised by median (range); n is the number of subjects.

**Table 5:** Overview of the proposed weight-banded dosing regimen for tideglusib in paediatric and adult patients with DM-1.

	Weight-Banded Dosing Regimen (mg/kg)						
Weight-band (kg)	*≥5* *–* *<10*	*≥10* *–* *<15*	*≥15* *–* *<20*	*≥20* *–* *<35*	*≥35* *–* *<45*	*≥45* *–* *<60*	*60+*
Maintenance Dose (mg)	100	200	300	400	600	800	1000

## Data Availability

The data can be shared up on request.

## Supplementary Materials

The following supporting information can be downloaded at: https://www.mdpi.com/article/10.3390/pharmaceutics17081065/s1. Section S1. Inclusion and Exclusion Criteria (Study NP031112-07A03). Section S2. Inclusion and Exclusion Criteria (Study AMO-02-MD-2-001). Section S3. Bioanalytical Method (Study NP031112-07A03). Section S4. Bioanalytical Method (Study AMO-02-MD-2-001). Section S5. Supplementary tables and figures. Table S1. Demographic baseline characteristics of the population included in the pharmacokinetic analysis. Table S2. Predicted area under the concentration vs. time curve (AUC_0–24_) after oral administration of a weight-banded dosing regimen of tideglusib (maintenance phase). Table S3. Predicted peak concentration (C_max_) after oral administration of a weight-banded dosing regimen of tideglusib (maintenance phase). Table S4. Proposed lower weight-banded dosing regimen for tideglusib in paediatric and adult patients with DM-1. Table S5. Predicted area under the concentration vs. time curve (AUC_0–24_) after oral administration of a weight-banded dosing regimen of tideglusib (titration phase). Table S6. Predicted peak concentration (C_max_) after oral administration of a weight-banded dosing regimen of tideglusib (titration phase). Figure S1. Scatter plots of observed plasma tideglusib concentrations in elderly healthy subjects stratified by dose group in study NP031112-07A03. Figure S2. Scatter plots of observed plasma tideglusib concentrations in adolescents and adults DM-1 patients enrolled in study AMO-02-MD2-001. Figure S3. Predicted tideglusib concentration vs. time profiles in elderly healthy subjects. Figure S4. Goodness of fit for the final pharmacokinetic model in elderly healthy subjects (study NP031112-07A03). Figure S5. NPDE results for the final model in elderly healthy subjects (study NP031112-07A03). Figure S6. Predicted tideglusib concentration vs. time profiles in adolescent and adult DM-1 patients following oral administration of a 400 or 1000 mg dose. Figure S7. Goodness of fit for the final pharmacokinetic model in adolescent and adult DM-1 patients (study AMO-02-MD2-001). Figure S8. NPDE results for the final model in adolescent and adult DM-1 patients (study AMO-02-MD2-001). Figure S9. Mirror plots. Individual predicted vs. observed tideglusib concentrations from the simulated data using the final model. Figure S10. Final model: distribution of weighted residuals and conditional weighted residuals. Figure S11. Correlations between the inter-individual random effects. Figure S12. Predicted C_max_ (upper panel) and AUC_0–24_ (lower panel) in children and adults stratified by bodyweight and receiving the proposed lower weight-band dosing regimen (titration dosing).
