# Supplementary material for: Population Pharmacokinetics of Tideglusib in Congenital and Childhood Myotonic Dystrophy Type 1: Influence of Demographic and Clinical Factors on Systemic Exposure
Source: Pharmaceutics. 2025 Aug 16;17(8):1065. doi: 10.3390/pharmaceutics17081065 (PMC12389255; doi:10.3390/pharmaceutics17081065)
Supplement: Supplementary file 1 [file pharmaceutics-17-01065-s001.zip › pharmaceutics-3503192-supplementary.pdf]

Supplementary Materials

Population Pharmacokinetics of Tideglusib in Congenital and Childhood Myotonic Dystrophy Type 1: Influence of Demographic and Clinical Factors on Systemic Exposure

Alessandro Di Deo [1,†], Sean Oosterholt [1,†], Joseph Horrigan [2], Stuart Evans [2] Alison McMorn [2] , and Oscar Della Pasqua [1*]

[1] Clinical Pharmacology & Therapeutics Group, University College London, UK

[2] AMO Pharma Ltd, Godalming, Surrey, UK

[†] These authors contributed equally to this work

* Correspondence: o.dellapasqua@ucl.ac.uk

Section S1. Inclusion and Exclusion Criteria (Study NP031112-07A03)

Inclusion Criteria
1. Healthy male and female subjects as determined by medical history, physical examination including vital signs, ECG recordings and clinical laboratory test results.
2. Age ≥ 60 years.
3. Body mass index (BMI) between 18 and 30 kg/m2 inclusive, with a body weight between 50 and 100 kg.
4. Negative serology: HIV-1/2 antibody, Hepatitis B surface antigen, Hepatitis C antibody.
5. Negative urine drug screen for cannabis, opiates, methadone, cocaine metabolite, amphetamines, barbiturates, benzodiazepines or other drugs of abuse, negative alcohol urine test.
6. Normal liver function test at Screening with values of ALT, AST, GGT, AP and bilirubin within normal reference ranges.
7. Subjects have to give their signed Informed Consent before any study-related activity.
8. Females participating in the study must be postmenopausal with no menstrual bleeding for at least 2 years prior the study.

Exclusion Criteria
1. Any underlying condition requiring the regular use of any medication.
2. Exposure to any medication, including over-the counter medications, 14 days prior to randomisation (except paracetamol).
3. Exposure to prescription or other drugs known to interfere with metabolism of drugs within 30 days prior to Screening.
4. Participation in another study with any investigational drug in the three months preceding the study.
5. Within the exclusion period of any preceding study.
6. Treatment in the previous three months with any drug known to have a well-defined potential for toxicity to a major organ.
7. Symptoms of a clinically significant illness within three months before the study.
8. Presence or sequelae of gastrointestinal, liver or kidney disease or other conditions known to interfere with the absorption, distribution, metabolism or excretion of drugs.
9. History of hypersensitivity to drugs with a similar chemical structure.
10. History of significant allergic disease and acute phase of allergic rhinitis in the previous two weeks before randomization or food allergy.
11. Blood or plasma donation of more than 500 mL during the previous 3 months before allocation and more than 50 mL in the two weeks before allocation on Day –1.
12. Smokers of more than five cigarettes/day or equivalent during the previous three months; or subjects unable to abstain from smoking during the in-house stay in the CPRU.
13. Current evidence of drug abuse or history of drug abuse within one year of randomization; positive urine drug screen.
14. History of alcohol abuse (i.e.: consumption >15 g/day) or active alcoholism; positive alcohol urine test.
15. Shift-worker unable to comply with the study.
16. Subjects who cannot abstain from strenuous physical exercise within 72 hours before Screening and before Day -1 until Follow-up.

17. Inability to understand the protocol requirements, instructions and study-related restrictions, the nature, scope, and possible consequences of the study.
18. Unlikely to comply with the protocol requirements, instructions and study-related restrictions; e.g., uncooperative attitude, inability to return for Follow-up visits, and improbability of completing the study.
19. Subject is the investigator or any sub-investigator, research assistant, pharmacist, study coordinator, other staff or relative thereof directly involved in the conduct of the study.
20. Vulnerable subjects (e.g. persons kept in detention).
21. Subjects who were administered NP031112 in a previous clinical study and reported any AE including clinically significant safety laboratory findings. Subjects who were in this regard inconspicuous in a previous study are not excluded from the present study.

Section S2. Inclusion and Exclusion Criteria (Study AMO-02-MD-2-001)

Inclusion Criteria

1. Adolescents or adults with diagnosis of congenital or juvenile-onset type 1 myotonic dystrophy (DM-1).
2. Diagnosis must be genetically confirmed.
3. Subjects must be male or female aged 12 years to 45 years.
4. Subjects must have a Clinical Global Impression - Severity (CGI-S) score of 4 or greater at Screening and Run-in (V2).
5. Subjects must be ambulatory and able to complete the 10 metre walk/run test (splints allowed).
6. Subject's legally authorized representative (LAR) must provide written informed consent and there must be written consent or assent (as age applicable and developmentally appropriate) by the subject before any study-related procedures are conducted.

Exclusion Criteria

1. Non-ambulatory (full time) wheelchair user.
2. Receiving stimulant medication.
3. Receiving other medications/therapies not stable (changed) within 4 weeks prior to Run-in (V2).
4. Medical illness or other concern which would cause investigator to conclude subjects will not be able to perform the study procedures or assessments or would confound interpretation of data obtained during assessment.
5. Current enrolment in a clinical trial of an investigational drug or enrolment in a clinical trial of an investigational drug in the last 6 months.
6. Women of childbearing potential who are pregnant, lactating or not willing to use a protocol defined acceptable contraception method if sexually active and not surgically sterile.
7. Gastrointestinal disease which may interfere with the absorption, distribution, metabolism or excretion of the study medication and impact the interpretability of the study results.
8. Current clinically significant (as determined by the investigator) cardiovascular, renal, hepatic, endocrine or respiratory disease.
9. Clinically significant heart disease (in the opinion of the investigator) or second or third degree heart block, atrial flutter, atrial fibrillation, ventricular arrhythmias, or is receiving medication for treatment of a cardiac arrhythmia.

10. A history of chronic liver disease with current out of range values for Alanine transaminase (ALT), clinically relevant hepatic steatosis or other clinical manifestations of ongoing liver disease.
11. A history of significant drug allergy (such as Steven-Johnson syndrome, anaphylaxis).
12. A history of alcohol or substance use disorders.

Section S3. Bioanalytical Method (Study NP031112-07A03)

Sample Collection

Human plasma samples obtained from the Phase I Clinical Trial NP031112-07A03 were sent by Parexel to Harlan Laboratories S.A., according to the Study Plan and the Clinical Trial Protocol requirements. Blood samples of 5.5 mL were collected into lithium heparin tubes and immediately put on ice. The samples were centrifuged within 20 min after collection at 3000 rpm for 10 min at approximately 4 ºC. The plasma was then immediately divided into two aliquots of at least 1.2 mL and transferred into plain in screw capped polypropylene tubes for storage at -80 ± 5 °C at PAREXEL awaiting shipment. All procedures between the sample collection and freezing were not to take more than 40 minutes. Once the plasma samples were received at Harlan Laboratories S.A., they were immediately stored at a nominal temperature of –80 ± 10 ºC until their analysis.

Quantitative analysis was performed by HPLC-MS/MS using collision induced dissociation. The bioanalytical HPLC-MS/MS methods used for the determinations were validated in the Study S01091. The long-term stability in spiked human plasma stored at -80 ± 10 ºC was evaluated in Study S03273. The lower limits of quantification (LLOQ) were set at 1 ng/mL in human plasma. The sample analysis lasted 3 minutes and mass spectrometry was operated in the MRM mode after collision induced dissociation.

Preparation of standard solutions

Two independent stock solutions of tideglusib were prepared. Stock solution A and its derived working solutions were used to prepare the matrix-based calibration standards (blank plasma samples spiked with a known amount of tideglusib) which will constitute the calibration curve. The quality control (QC) samples were prepared from stock solution B and its derived working solutions. The concentration of NP031112 present in the test and QC samples was therefore quantified by using the calibration curve prepared from solution A.

Tideglusib was accurately weighed and dissolved with MeOH/ACN (1:1, v/v) in a 10-mL volumetric flask to give a final concentration of 1 mg of tideglusib/mL. Stock solutions A and B were stored at -20 ± 5 ºC. The stock solutions were aliquoted. Each aliquot was stored at -20 ± 5 ºC for a maximum storage period of 7 days. One aliquot of the stock solution to be used was thawed daily in order to prepare the corresponding working solutions as detailed below. The employed aliquot was discarded and not re-used.

Working solutions of 40, 20, 5, 1, 0.2, and 0.04 µg/mL were prepared from stock solutions A and 20, 1, and 0.04 µg/mL from stock solution B. These working solutions were prepared in volumetric flasks as follows:

- A working solution of 40 µg/mL was prepared by diluting 200 µL from the stock solution up to 5 mL with the solution MeOH / ACN / Milli-Q grade water (1:1:2, v/v/v).
- A working solution of 20 µg/mL was prepared by diluting 100 µL from the stock solution up to 5 mL with the solution MeOH / ACN / Milli-Q grade water (1:1:2, v/v/v).

- A working solution of 5 μg/mL was prepared by diluting 50 μL from the stock solution up to 10 mL with the solution MeOH / ACN / Milli-Q grade water (1:1:2, v/v/v).
- A working solution of 1 μg/mL was prepared by diluting 500 μL from the 20 μg/mL solution up to 10 mL with the solution MeOH / ACN / Milli-Q grade water (1:1:2, v/v/v).
- A working solution of 0.2 μg/mL was prepared by diluting 100 μL from the 20 μg/mL solution up to 10 mL with the solution MeOH / ACN / Milli-Q grade water (1:1:2, v/v/v).
- A working solution of 0.04 μg/mL was prepared by diluting 200 μL from the 1 μg/mL solution up to 5 mL with the solution MeOH / ACN / Milli-Q grade water (1:1:2, v/v/v).

Calibration curves

Eventually, for the characterisation of the calibration curve, plasma from untreated volunteers was spiked with various amounts of tideglusib. The calibration curves used for the quantification of tideglusib in the quality control samples were prepared from stock solution A and its derived working solutions. For each compound, 6 quality control samples were prepared from stock solution B and its derived working solutions.

The correlation coefficients obtained for each calibration curve was higher than 0.99. 75% of the back-calculated concentrations did not deviate by more than 15% or 20% (LLOQ) of the nominal value.

Chromatographic conditions

The column used was a HPLC column (Atlantis® dC RP18, 3.0 μm particle size, 100 mm x 4.6 mm ID, Waters) with a HPLC pre-column (X-Terra® RP18, Waters). The column temperature was set at 40°C, while the injector temperature was maintained at 4°C. The run-time was 3 minutes, and the mobile phase consisted of MeOH/ACN/0.1% Formic acid in a ratio of 85:5:10 (v/v/v). The retention time was approximately 2.00 minutes. The mass transaction used was m/z 333.97 > m/z 273.25, with a dwell time of 0.3 seconds.

Section S4. Bioanalytical Method (Study AMO-02-MD-2-001)

The bioanalytical procedures adopted for the determination of tideglusib in DM-1 patients are based on the study S56195. The study aimed to partially validate the bioanalytical methods for the determination of tideglusib and its main metabolite, NP04113, in human plasma using lithium heparin as anticoagulant and to demonstrate the stability of tideglusib and NP04113 in plasma samples following two different freezing ways at -80 ± 10 ºC. The bioanalytical methods are based on LC-MS/MS methods developed, validated and revalidated at Envigo CRS, S.A.U. (Study Numbers: CD05/9560FC, S01091, S03273, S09988, S26427, S29871 and S56184).

Bioanalytical methods for the determination of tideglusib and NP04113 were validated at Harlan Laboratories S.A. in GLP Study S01091 (Harlan, 2007) and developed at Envigo CRS, S.A.U. in non-GLP Study S56184. NP04113 is the urea derivate of tideglusib and is presumed to be generated from tideglusib by in vivo biotransformation processes.

Tideglusib extraction method consists in a solid-phase extraction involving a hydrophilic/lipophilic balanced sorbent using dichloromethane solution to elute and after a treatment of the plasma with a saturated solution of ammonium chloride at pH 9.5 adjusted with 35% ammonia solution. The dry residue is dissolved in methanol / acetonitrile / Milli-Q grade water pH 2.2 (85:5:10, v/v/v). The final extract of the prepared samples for the upper range is diluted 40-fold in order to avoid response saturation. Finally, 20 μL are injected into an LC-MS/MS system.

These products were determined by the AcquityTM UPLC® system with tandem mass spectrometry in the positive electrospray ionization mode. The retention time of Tideglusib under chromatographic conditions used were ≈ 0.48 min with sample analysis completed within 3 minutes. The best mass transition obtained after collision-induced dissociation and used for quantitative purpose is *m/z* 335.18 > *m/z* 91.09.

The validation of the bioanalytical method for tideglusib in human plasma demonstrated compliance with regulatory requirements. The method covers a concentration range of 1–2000 ng/mL, with an LLOQ of 1 ng/mL. Accuracy for the lower and upper range were [91.20% to 109.03%] and [84.13% to 108.83%] and [99.71% to 101.99%] and [97.93% to 101.73%] for intra- and inter-assay measurements. Precision for the lower and upper range were [2.38% to 12.59%] and [0.85% to 5.5%] and [8.06% to 9.66%] and [4.21% to 10.87%] for intra- and inter-assay measurements. Stability assessments confirmed sample integrity over 208 days at -80°C, with freeze/thaw stability for three cycles. The method showed no significant carry-over, and processed samples remained stable for up to four days at 2–8°C. A dilution factor up to 10x was validated. The quantification transition used for tideglusib was **m/z** 335.18 → **m/z** 91.09.

Human plasma, containing lithium heparin as anticoagulant, was obtained from untreated volunteers within Hospital Sant Pau and stored at -20 ± 5 ºC when not in use. Pooled plasma was used except for the control matrix source test and for the matrix factor sample preparation where six independent sources (individual) were used.

Human haemolysed plasma, containing lithium heparin as anticoagulant, was prepared from fresh whole blood by using saponin. Whole blood was obtained from untreated volunteers within Hospital Sant Pau and was received refrigerated. The blank plasma has been stored at -20 ± 5 ºC until use for no more than two years.

Section S5. Supplementary tables and figures

Table S1 Demographic baseline characteristics of the population included in the pharmacokinetic analysis

	Dose Group (mg)	Dosing regimen	Subjects (n/female)	Body weight (kg)	Height (cm)	Age (years)
NP03112-07A03	300.	b.i.d.	9/4	74.8 [10.1]	170.0 [7.3]	63.0 [3.2]
	400	b.i.d.	9/5	76.2 [12.6]	168.8 [11.0]	67.4 [3.4]
	600	q.d.	9/4	76.8 [7.6]	171.2 [7.3]	64.3 [3.5]
	800	q.d.	9/5	76.5 [6.4]	169.8 [8.2]	65.4 [3.9]
	1000.	q.d.	9/2	71.3 [11.0]	169.3 [9.8]	62.4 [1.9]
	1200	q.d.	9/4	71.5 [12.0]	167.1 [8.4]	63.6 [3.2]
AMO-02 MD-2-001	400.	q.d.	8/1	59.9 [12.6]	166.3 [8.3]	20.2 [6.4]
	1000.	q.d.	8/5	67.4 [26.7]	163.1 [10.5]	21.7 [5.5]

Values are mean [±SD]

Table S2 Predicted area under the concentration vs time curve (AUC_{0-24}) after oral administration of weight-banded dosing regimen of tideglusib (maintenance phase)

Body weight (kg)	Dose (mg)	AUC_{0-24} (ng/mL.h)		
		Median (5th – 95th percentiles)	*Mean (±SD)*	*Subjects above the reference threshold* (%)*
5 - <10	100	1672.9 (760.3-3549.7)	1857.0 (±900.6)	0.62%
10 - <15	200	2237.2 (1045.2-4622.8)	2450.3 (±1155.3)	2.72%
15 - <20	300	2558.6 (1226.9-5249.2)	2805.4 (±1274.6)	5.16%
20 - <35	400	2398.9 (1144.6-5077.3)	2666.0 (±1282.7)	4.42%
35 - <45	600	2363.8 (1161.5-4923.9)	2621.8 (±1216.7)	3.67%
45 - <60	800	2579.0 (1261.6-5233.1)	2827.6 (±1294.2)	5.10%
60 - <70	1000	2766.8(1339.5-5550.1)	3026.3 (±1367.0)	6.87%
70-75 (ref.)	1000	2523.8 (1243.7-5213.3)	2779.1 (±1265.7)	5.00%

* The threshold represents the 95th percentile (5213.3 ng/mL.h) of the reference range for the adult population (70-75 kg) receiving a 1000 mg dose of tideglusib.

Table S3 Predicted peak concentration (C_{max}) after oral administration of weight-banded dosing regimen of tideglusib (maintenance phase)

Body weight (kg)	Dose (mg)	C_{max} (ng/mL)		
		Median (5th – 95th percentiles)	*Mean (±SD)*	*Subjects above the reference threshold* (%)*
5 - <10	100	717.7 (312.2-1503.4)	792.7 (±385.3)	2.04%
10 - <15	200	914.6 (411.6-1853.1)	997.3 (±451.8)	6.10%
15 - <20	300	1039.7 (477.9-2053.0)	1126.0 (±499.4)	10.32%
20 - <35	400	947.4 (434.0-1912.3)	1033.7 (±472.8)	7.02%
35 - <45	600	912.2 (429.4-1772.8)	981.9 (±429.3)	4.97%
45 - <60	800	970.9 (457.3-1895.9)	1045.8 (±454.2)	6.94%
60 - <70	1000	1024.5 (486.5-1954.0)	1095.0 (±460.5)	8.13%
70-75 (ref.)	1000	921.5 (445.1-1774.7)	994.6 (±424.1)	5.00%

* The threshold represent the 95th percentile (1774.7 ng/mL) of the reference range for the adult population (70-75 kg) receiving a 1000 mg dose of tideglusib.

Table S4 Proposed lower weight-banded dosing regimen for tideglusib in paediatric and adult patients with DM-1

	Weight-banded dosing regimen (mg/kg)						
Weight-band (kg)	*≥5 and <10*	*≥10 and <15*	*≥15 and <20*	*≥20 and <35*	*≥35 and <45*	*≥45 and <60*	*60+*
Titration Dose (mg)	100	100	200	200	200	400	400

Table S5 Predicted area under the concentration vs time curve (AUC_{0-24}) after oral administration of weight-banded dosing regimen of tideglusib (titration phase)

Body weight (kg)	Dose (mg)	AUC_{0-24} (ng/mL.h)		
		Median (5th – 95th percentiles)	*Mean (±SD)*	*Subjects above the reference threshold* (%)*
5 - <10	100	1672.9 (760.3-3549.7)	1857.0 (±900.6)	0.62%
10 - <15	100	1118.7 (522.6-2311.5)	1225.2 (±577.6)	0.02%
15 - <20	100	852.9 (409.0-1749.7)	935.1 (±424.9)	0.00%
20 - <35	200	1199.5 (572.3-2538.6)	1333.0 (±641.4)	0.06%
35 - <45	200	894.4 (439.7-1864.1)	992.1 (±460.7)	0.00%
45 - <60	400	1463.8 (715.4-2971.7)	1604.9 (±735.0)	0.20%
60 - <70	400	1255.7 (608.0-2520.7)	1374.1 (±621.1)	0.04%
70-75 (ref.)	400	1145.1 (564.1-2366.7)	1261.9 (±575.1)	0.02%

* The threshold represents the 95th percentile (5213.3 ng/mL.h) of the reference range for the adult population (70-75 kg) receiving a 1000 mg dose of tideglusib.

Table S6 Predicted peak concentration (C_{max}) after oral administration of weight-banded dosing regimen of tideglusib (titration phase)

Body weight (kg)	Dose (mg)	C_{max} (ng/mL)		
		Median (5th – 95th percentiles)	*Mean (±SD)*	*Subjects above the reference threshold* (%)*
5 - <10	100	717.7 (312.2-1503.4)	792.7 (±385.3)	2.04%
10 - <15	100	457.3 (205.8-926.6)	498.7 (±225.9)	0.00%
15 - <20	100	346.6 (159.3-684.3)	375.3 (±166.5)	0.00%
20 - <35	200	473.7 (217-956.2)	516.8 (±236.4)	0.10%
35 - <45	200	345.4 (162.5-671.5)	371.9 (±162.6)	0.00%
45 - <60	400	551.5 (259.8-1077.0)	594.1 (±258.1)	0.15%
60 - <70	400	465.6 (221.1-888.1)	497.6 (±209.3)	0.02%
70-75 (ref.)	400	418.8 (202.1-806.6)	452.0 (±192.8)	0.02%

* The threshold represents the 95th percentile (1774.7 ng/mL) of the reference range for the adult population (70-75 kg) receiving a 1000 mg dose of tideglusib.

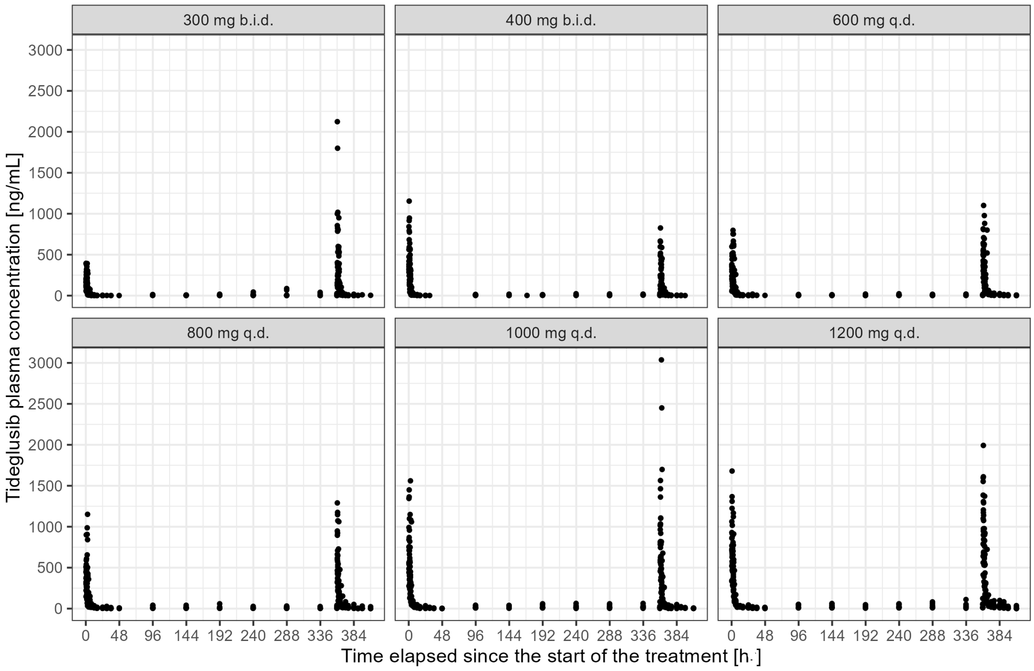

Figure S1. Scatter plots of observed plasma tideglusib concentrations in elderly healthy subjects stratified by dose group in study NP031112-07A03. Panels show observed concentrations (solid circles) versus time since the start of the treatment.

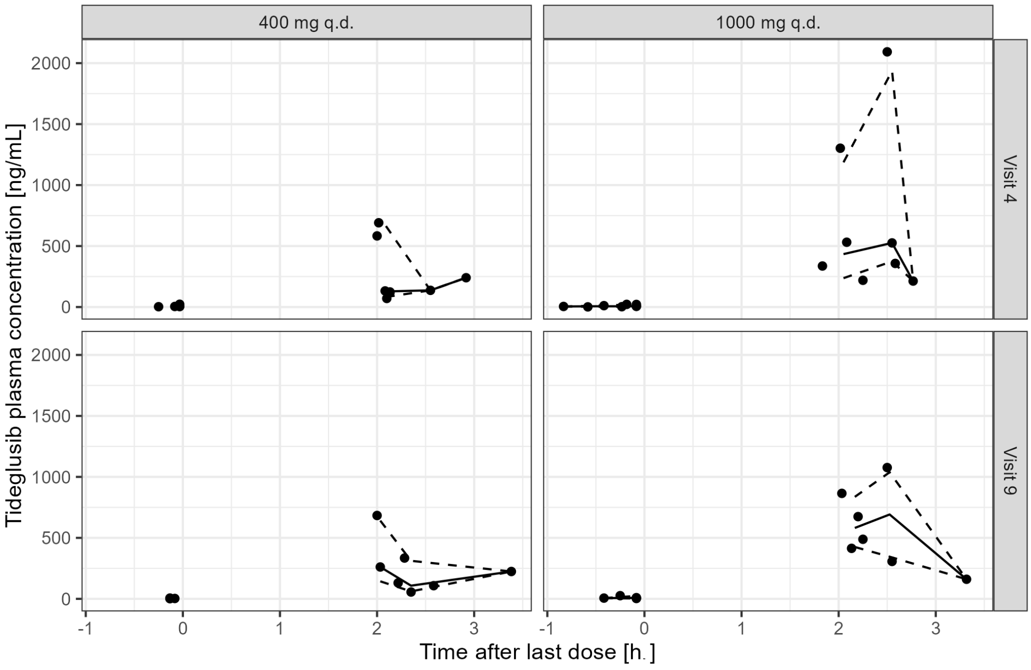

Figure S2. Scatter plots of observed plasma tideglusib concentrations in adolescents and adults DM-1 patients enrolled in study AMO-02-MD2-001. Panels show observed concentrations (solid circles) versus time after last dose stratified by visit. Solid and dashed lines represent, respectively, median, 5th and 95th percentiles.

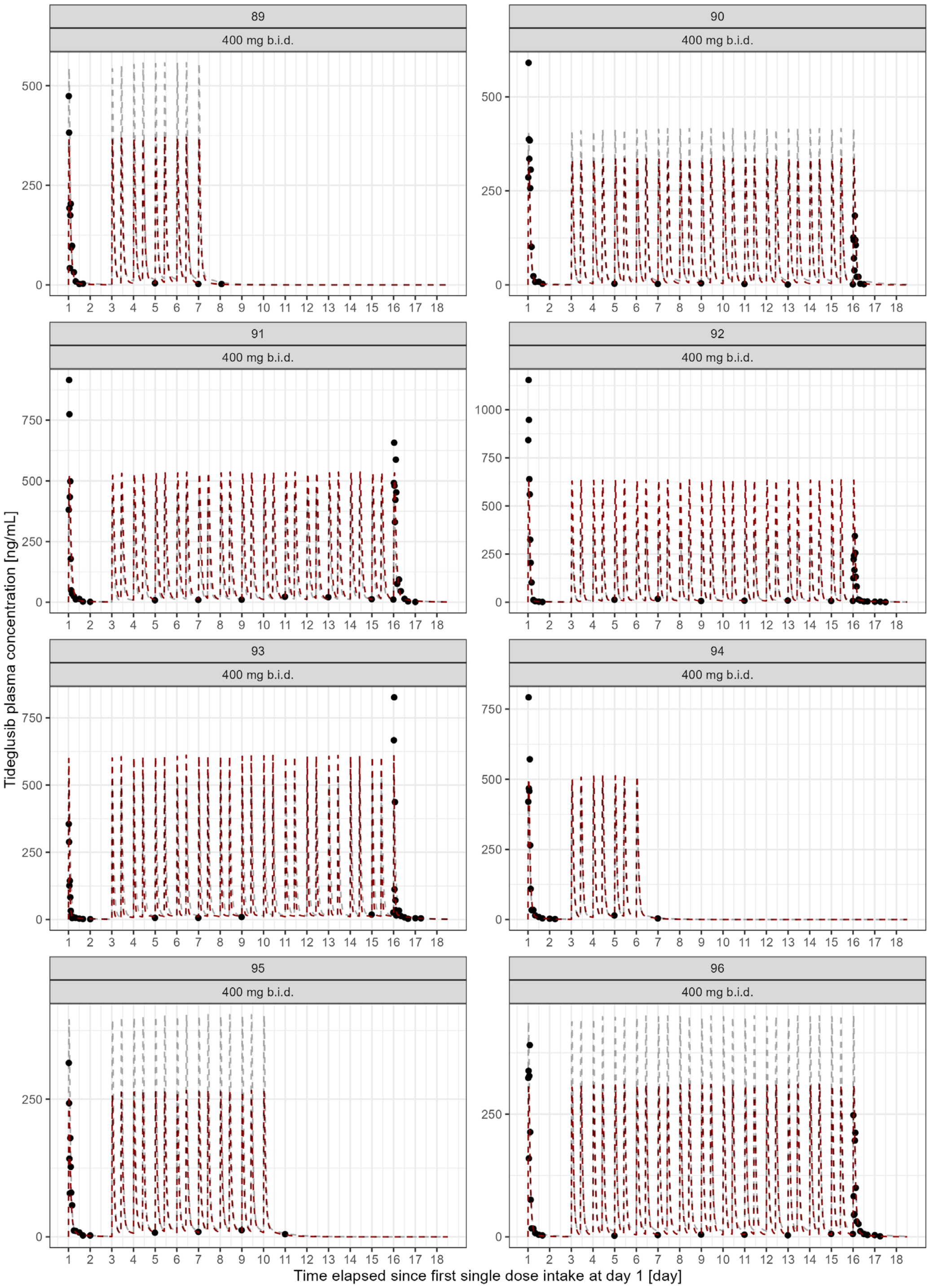

Figure S3. Predicted tideglusib concentration vs. time profiles in elderly healthy subjects. Graphs depict the first 8 subjects enrolled in the 400 mg b.i.d. treatment arm. Solid circles are observed concentrations. Red dashed lines indicate the individually predicted concentrations. Black dashed lines indicate the population predicted concentrations.

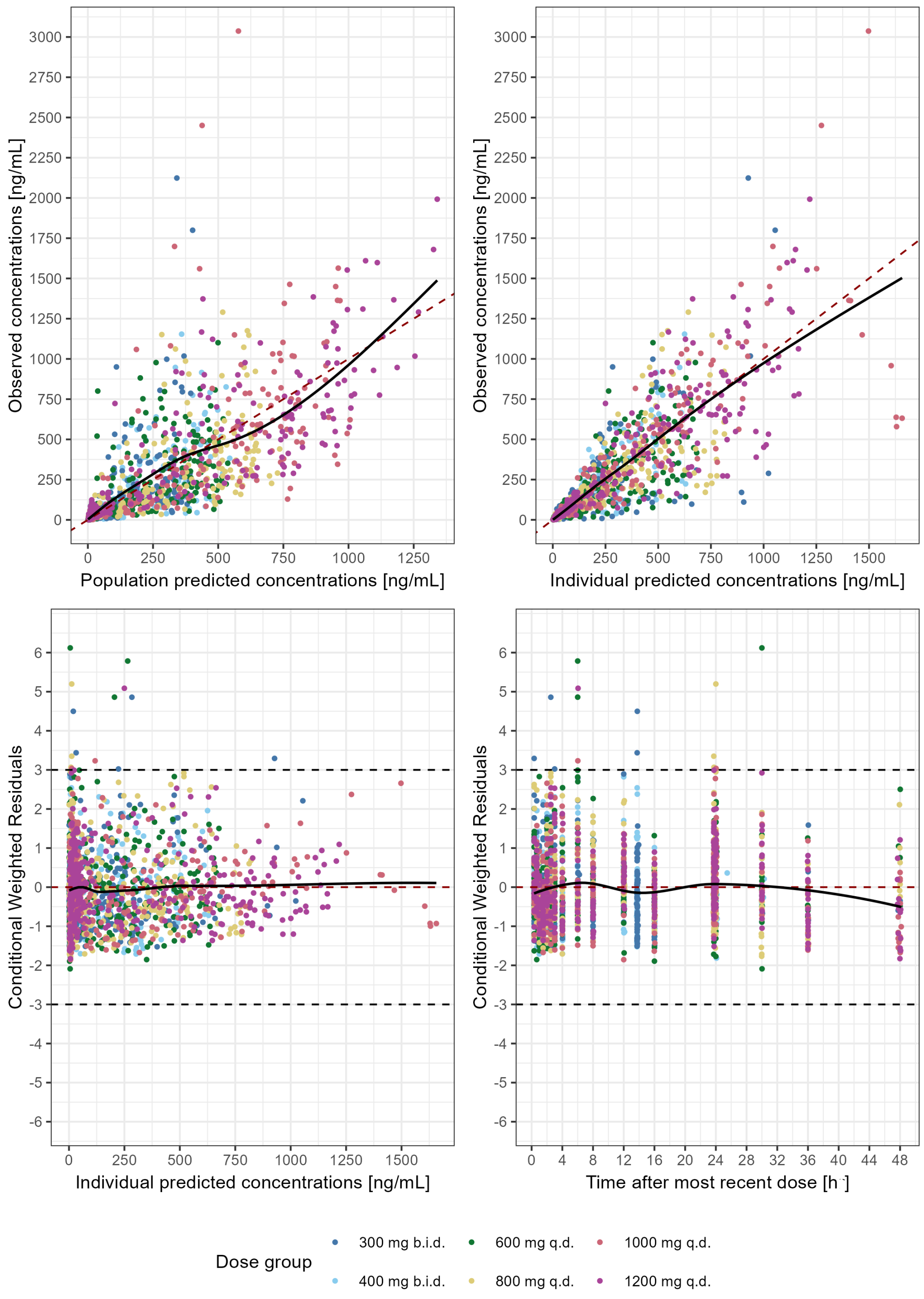

Figure S4. Goodness of fit for the final pharmacokinetic model in elderly healthy subjects (study NP031112-07A03). Upper panels show the observed vs. individual (left) and population (right) predicted concentrations; lower panels show conditional weighted residuals vs. individual predicted concentrations (left) and time (right). Solid circled are shown in colours to indicate the different dose groups; dashed red line depicts either the identity line in the upper panels or y = 0 in the lower panels, whereas the black solid line is the local polynomial regression.

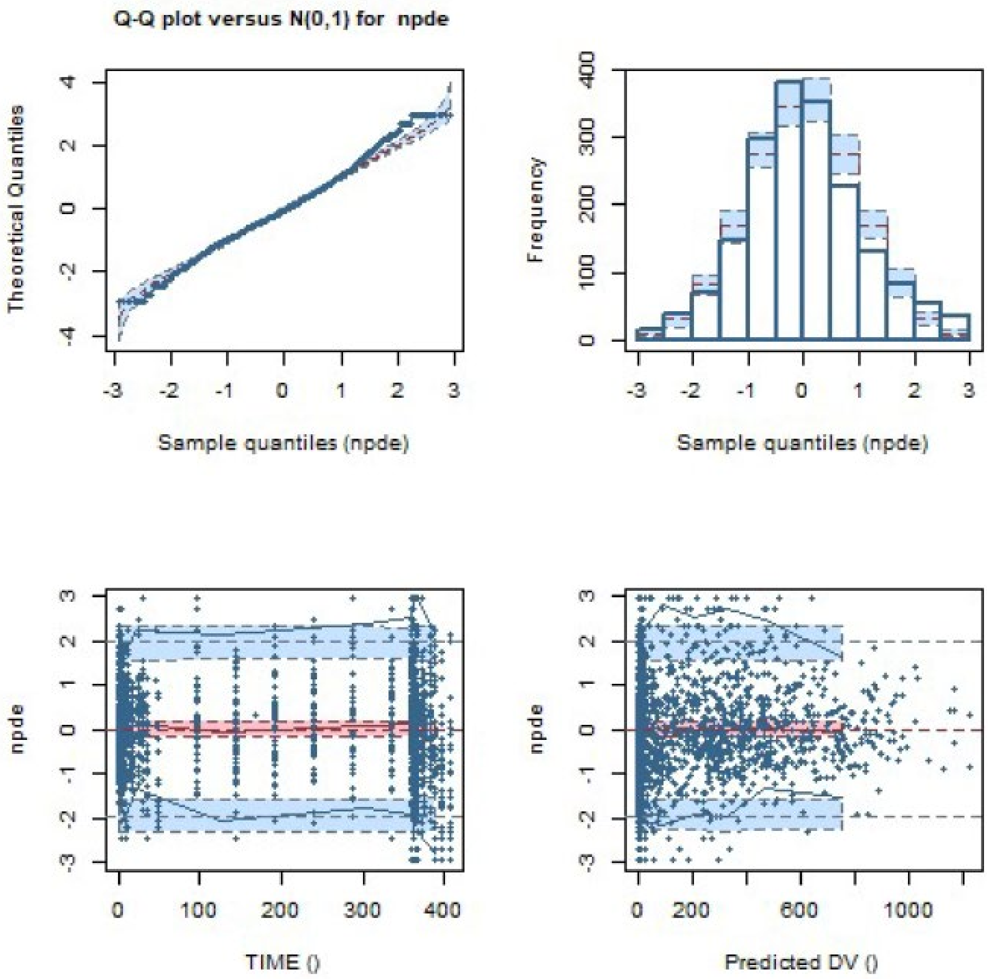

Figure S5. NPDE results for the final model in elderly healthy subjects (study NP031112-07A03). Top right: QQ-plot of the distribution of the NPDE versus the theoretical N (0,1) distribution. Top left: Histogram of the distribution of the NPDE, with the density of the standard Gaussian distribution overlaid. Bottom left: NPDE versus time. Bottom right: NPDE versus population predicted concentrations. Dashed lines in the top graphs represent the 95% prediction interval for a normal distribution. Dashed lines in the lower panels represent the 10th; 50th and 90th percentiles of the NPDE corresponding to observed data. Shaded areas represent the 95% prediction intervals for the 2.5th, 50th and 97.5th percentiles. Red lines/shaded area depict the median, whereas the blue lines/shaded area show the 2.5th and 97.5th quantiles of observed data. Solid blue circles indicate the individual observed values.

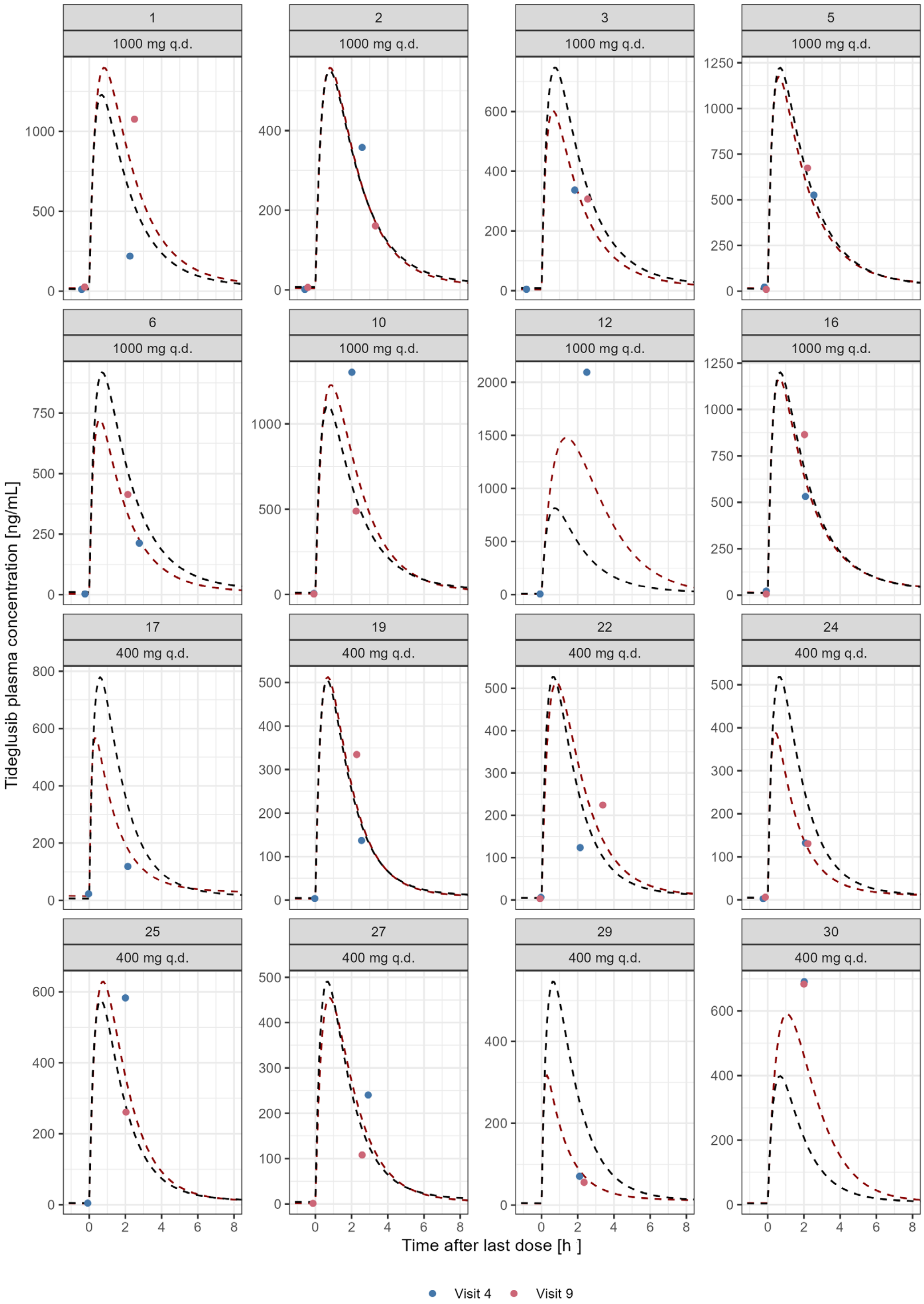

Figure S6. Predicted tideglusib concentration vs. time profiles in adolescent and adult DM-1 patients following oral administration of a 400 or 1000 mg dose. Solid circles are observed concentrations stratified by visit. Red dashed lines indicate the individually predicted concentrations. Black dashed lines indicate the population predicted concentrations.

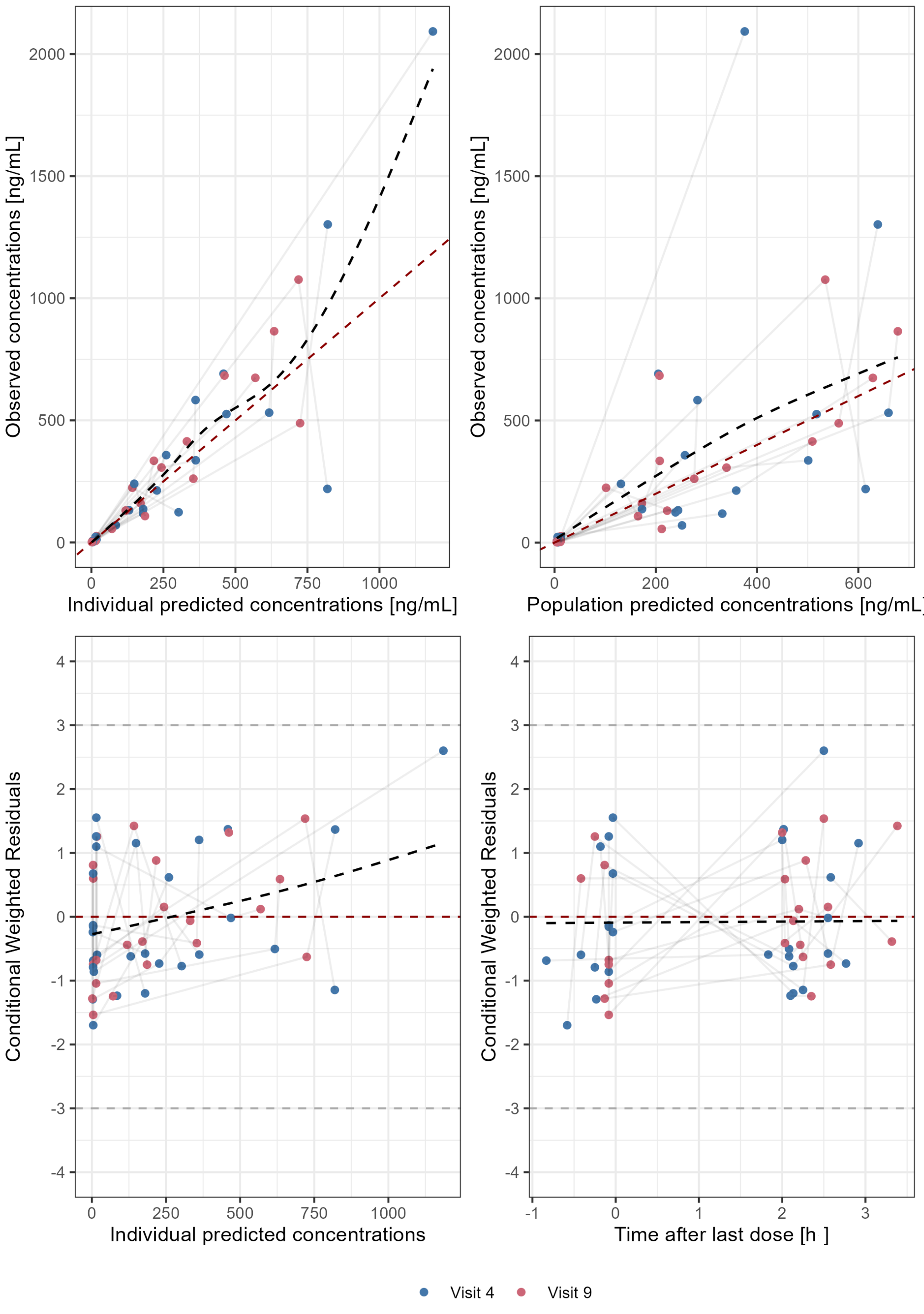

Figure S7. Goodness of fit for the final pharmacokinetic model in adolescent and adult DM-1 patients (study AMO-02-MD2-001). Upper panels show the observed vs. individual (left) and population (right) predicted concentrations; lower panels show conditional weighted residuals vs. individual predicted concentrations (left) and time (right). Solid blue and red circles represent individual observations/predictions for visits 4 and 9, respectively. Dashed red line depicts either the identity line in the upper panels or y = 0 in the lower panels. All observed and predicted concentrations of tideglusib are in ng/mL.

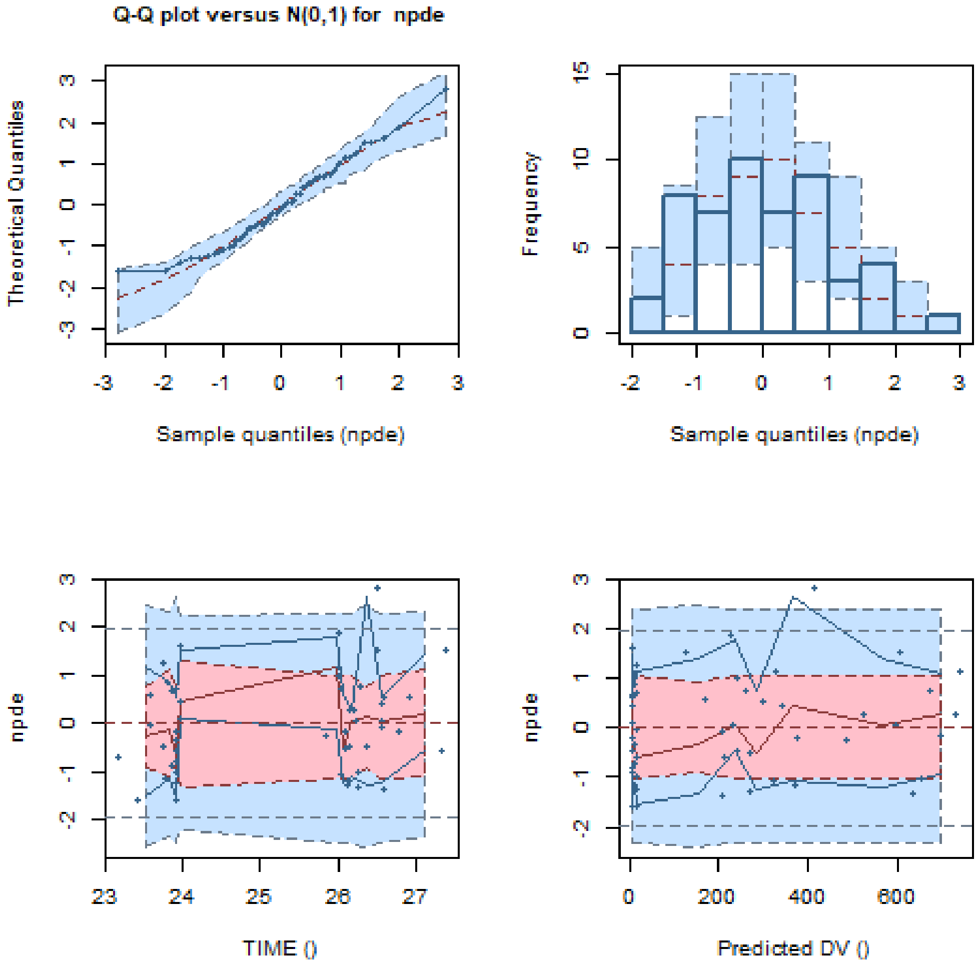

Figure S8. NPDE results for the final model in adolescent and adult DM-1 patients (study AMO-02-MD2-001). Top left: QQ-plot of the distribution of the NPDE versus the theoretical N (0,1) distribution. Top right: Histogram of the distribution of the NPDE, with the density of the standard Gaussian distribution overlaid. Bottom left: NPDE versus time. Bottom right: NPDE versus population predicted concentrations. Dashed lines in the top graphs represent the 95% prediction interval for a normal distribution. Dashed lines in the bottom graphs represent 10th; 50th and 90th percentiles of the NPDE corresponding to observed data. Shaded areas represent the 95% prediction intervals for the 2.5th, 50th and 97.5th percentiles. Red lines/shaded area depict the median, whereas blue lines/shaded area show the 2.5th and 97.5th quantiles of observed data. Blue dots indicate individual observed values.

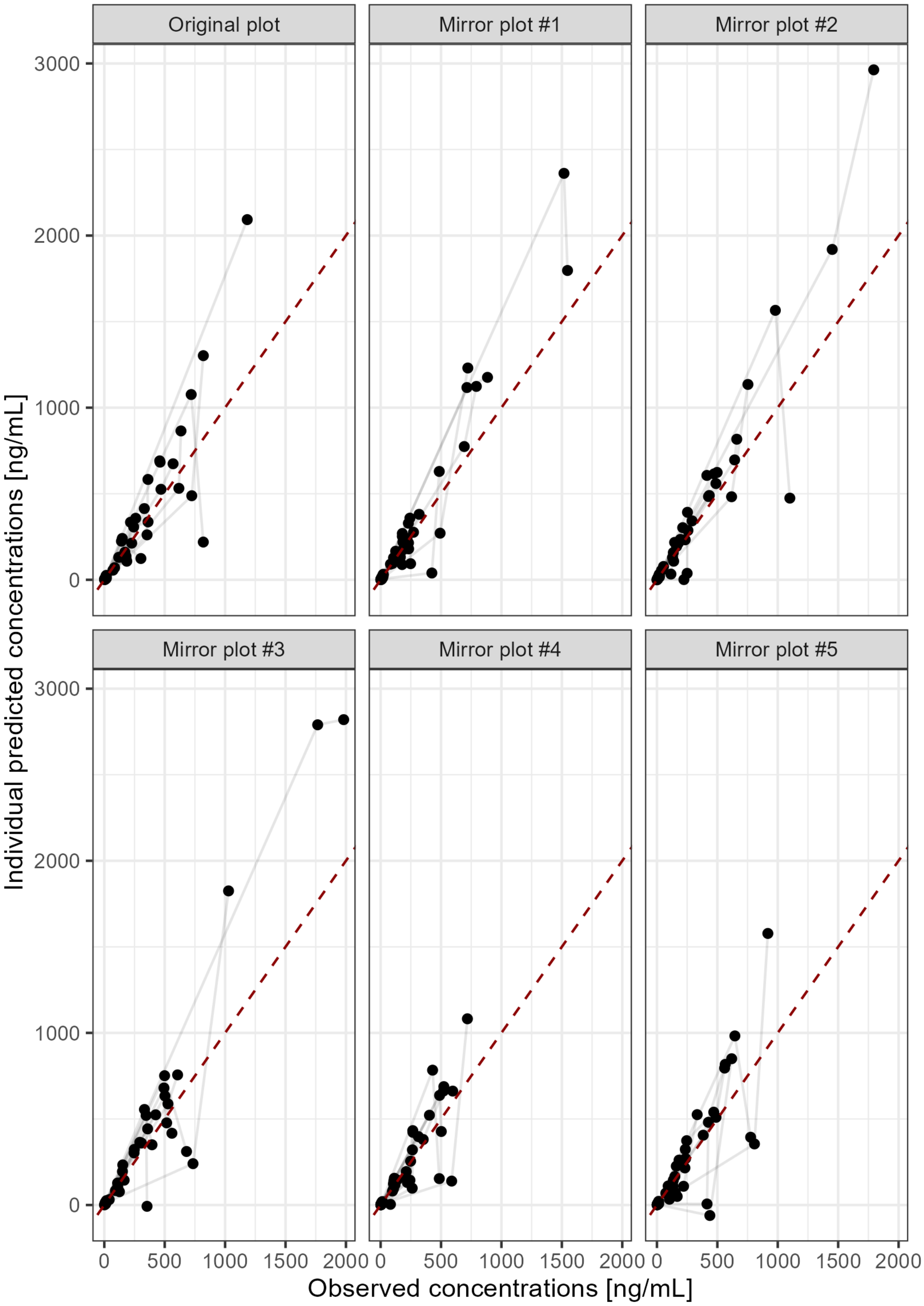

Figure S9. Mirror plots. Individual predicted vs observed tideglusib concentrations from the simulated data using the final model. Dashed line represents the identity line. Black dots and solid grey line represent the observed data in the original study along with model predictions from different simulations. In the top left panel are shown the original post-hoc individual predictions.

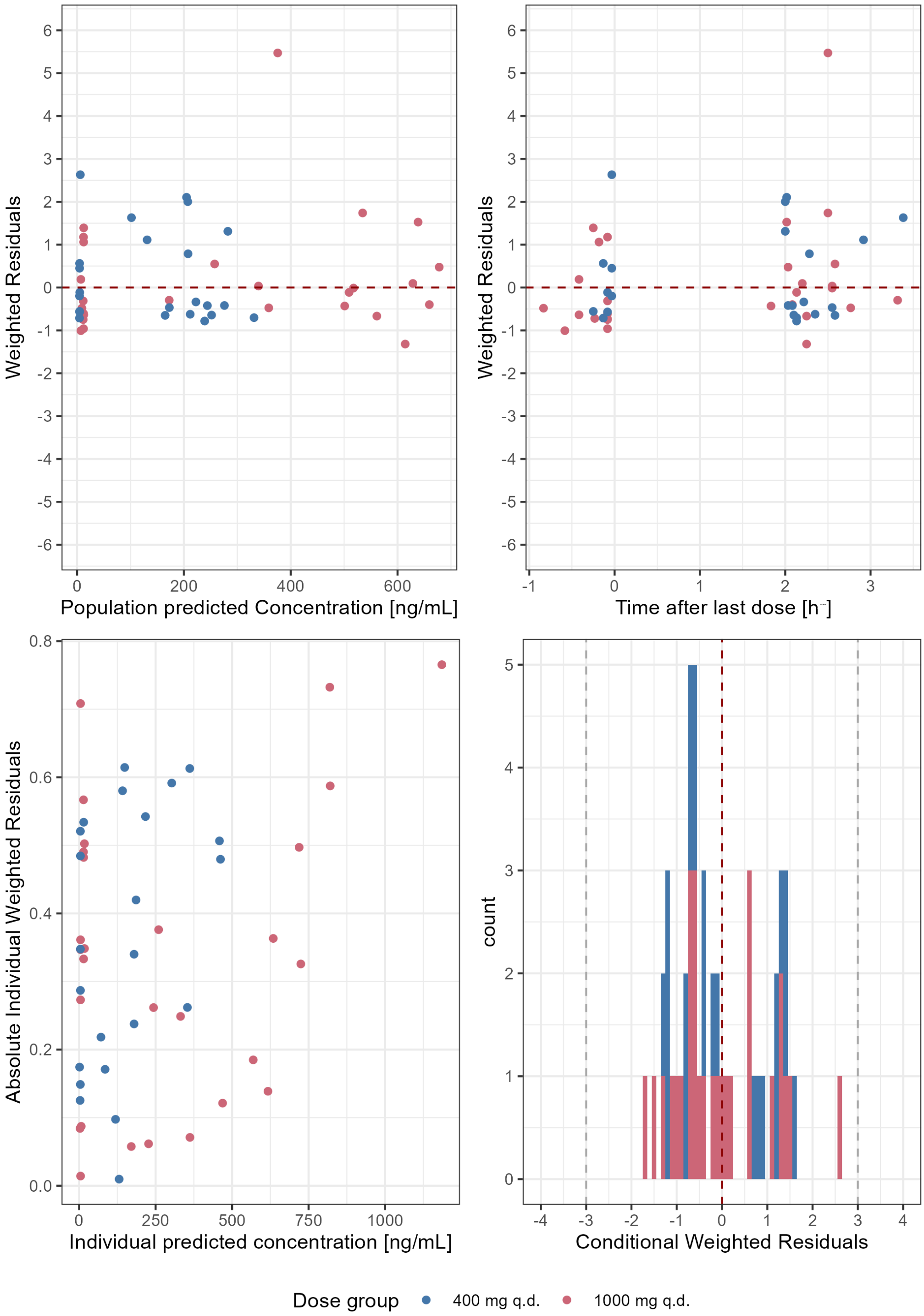

Figure S10. Final model: distribution of weighted residuals and conditional weighted residuals. Panels show weighted residuals vs. population predicted concentration (top left), time vs weighted residuals (top right), individual predicted concentrations vs absolute weighted residuals (bottom left), histogram of conditional weighted residuals (bottom right).

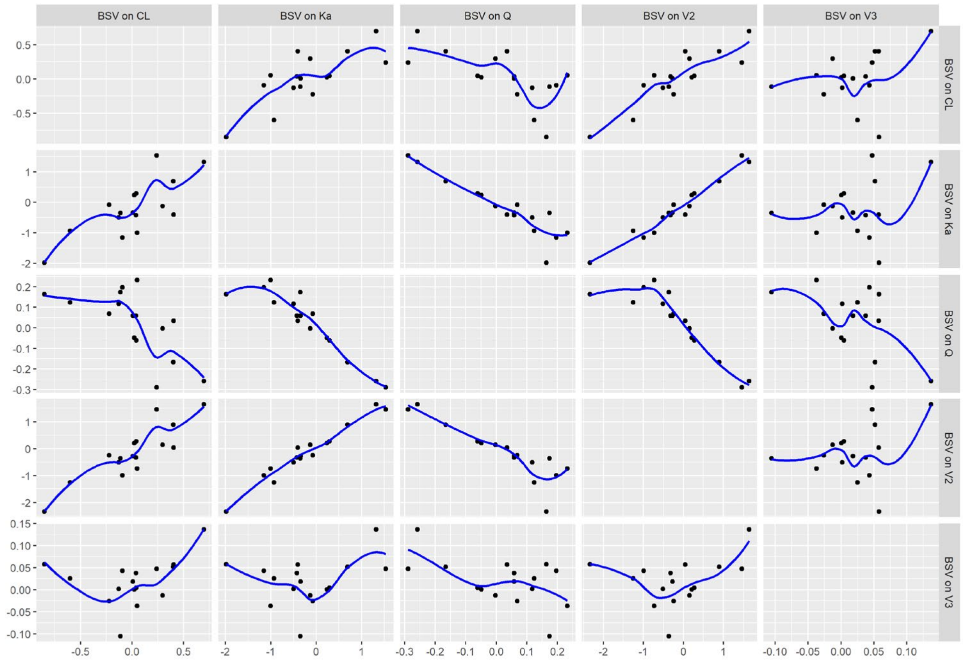

Figure S11. Correlations between the inter-individual random effects. Black circles indicate individual observed values for each of the covariates. The blue line is a general smoothing function, which indicates the presence of any trends. Each column and row describe a specific between-subject variability (BSV) component.

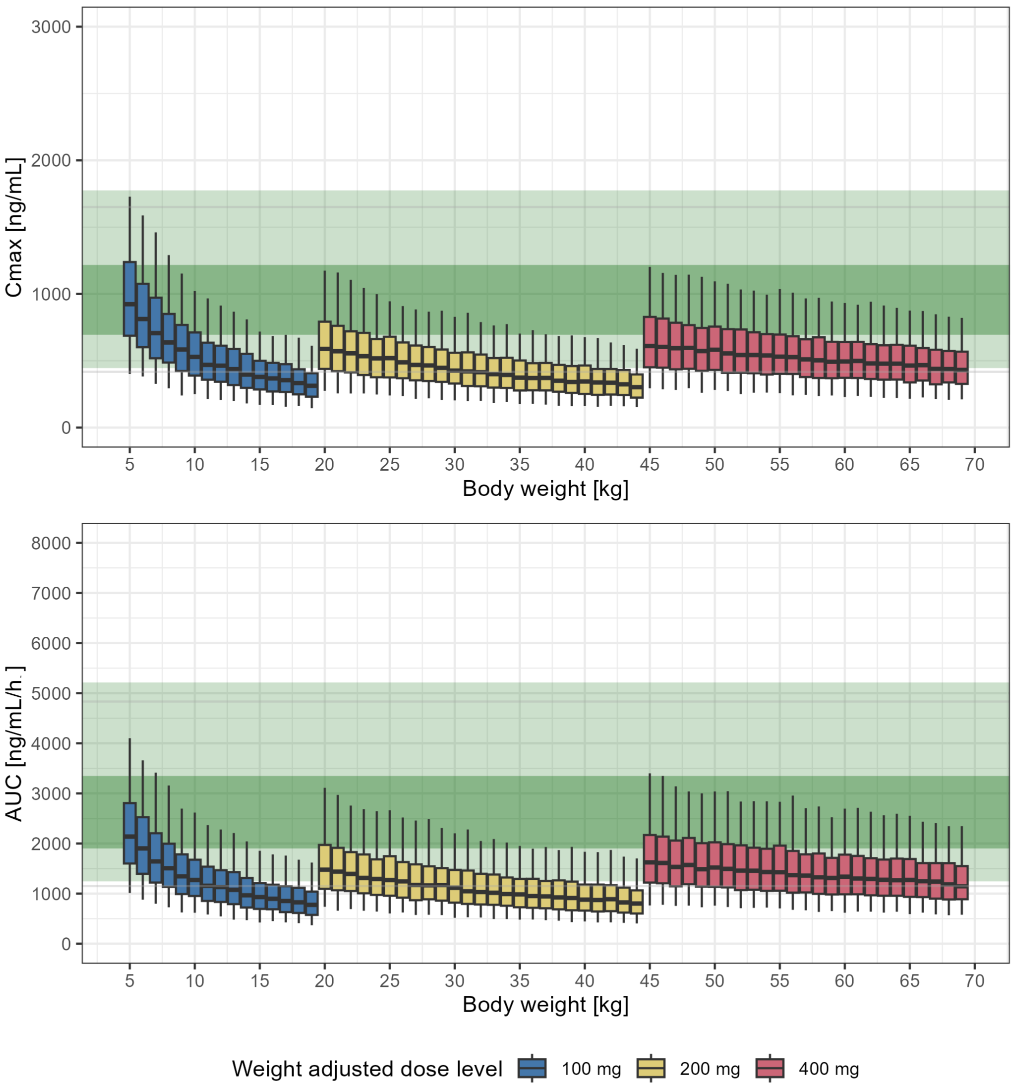

Figure S12. Predicted C_{max} (upper panel) and $AUC_{0\text{-}24}$ (lower panel) in children and adults stratified by body weight after administration of tideglusib according to a titration schedule. Shaded green area depict the reference exposure range (5th-95th and 25th-75th percentiles) predicted in adults with body weight equal to 70-75 kg following a 1000 mg dose of tideglusib. The horizontal line in the whisker-box plots represents the median, bottom and top edge of the box are the 25th and 75th percentiles. The vertical line (whiskers) represents the 5th and 95th percentiles of the data distribution. Each box refers to a specific weight group.
